# Capacity for Compensatory Cyclin D2 Response Confers Trametinib Resistance in Canine Mucosal Melanoma

**DOI:** 10.3390/cancers17142357

**Published:** 2025-07-15

**Authors:** Bih-Rong Wei, Vincenzo Verdi, Shuling Zhang, Beverly A. Mock, Heather R. Shive, R. Mark Simpson

**Affiliations:** 1 Laboratory of Cancer Biology and Genetics, Center for Cancer Research, National Cancer Institute, National Institutes of Health, Bethesda, MD 20892, USAvincenzo.verdi@nih.gov (V.V.); mockb@mail.nih.gov (B.A.M.);; 2 Leidos Biomedical Research Inc., 8560 Progress Drive, Frederick, MD 21702, USA

**Keywords:** targeted therapy resistance, molecular mechanism of drug resistance, small-molecule combination therapy, naturally occurring dog cancer, comparative oncology

## Abstract

**Background/objective:** Mucosal melanoma (MM) is a poorly responsive, rare and aggressive subtype with few cases having targetable recurrent driver mutations, although Ras/MAPK and PI3K/AKT/mTOR signaling pathway activations are common. Eventual tumor evasion of targeted therapy continues to limit treatment success. Adequate models are necessary to address therapeutic resistance. The relatively greater incidence of naturally occurring MM in dogs, as well as its comparable clinical and pathological characteristics to human MM, represents an opportunity for study as a human MM patient surrogate. Resistance-promoting crosstalk between Ras/MAPK and PI3K/AKT/mTOR signaling under trametinib inhibition of MEK was studied in canine MM. Emphasis was placed on the suppressive effect of trametinib on cell cycle entry and its potential role in drug resistance. **Methods**: D-type cyclins were investigated following trametinib treatment of five MM cell lines exhibiting differential drug sensitivities. Signaling pathway activation, proliferation, survival, cell death, and cell cycle were analyzed in the context of D-type cyclin expression. Cyclin D2 expression was manipulated using siRNA knockdown or inducible recombinant overexpression. **Results**: Trametinib diminished cyclin D1 in all cell lines. While relatively trametinib-resistant MM cells exhibited capacity to upregulate cyclin D2, which promoted proliferation, sensitive MM cells lacked similar cyclin D2 compensation. Inhibition of the compensatory cyclin D2 in resistant cells conferred sensitivity. Induced cyclin D2 overexpression in otherwise trametinib-sensitive MM cells promoted survival. Upregulated PI3K/AKT/mTOR signaling under trametinib treatment was suppressed by mTORC1/2 inhibition, which similarly diminished cyclin D2 response. **Conclusions**: The compensatory switch from preferential reliance on cyclin D1 to D2 plays a role in MM resistance to MEK inhibition.

## Simple Summary

Cancers can acquire resistance to treatments through multiple means, and therapeutic resistance continues to be a serious challenge to successful patient outcomes. Escape from cancer therapy is accompanied by continued tumor growth and possible metastasis in the most aggressive forms. For a better understanding of mucosal melanoma resistance to the MEK inhibitor, trametinib was studied by examining cell cycle-initiating cyclin D expression. Mucosal melanoma cancer cells capable of mediating the compensatory switch from cyclin D1 to cyclin D2, important molecules that cells employ to replicate, exhibited greater resistance to trametinib’s downstream suppressive effects on cyclin D1. Mucosal melanoma cells lacking sufficient cyclin D2 compensation were more trametinib-sensitive. Inhibiting the cyclin D subtype switch by targeting the PI3K/AKT/mTOR pathway in resistant cells helped maintain a better degree of drug sensitivity. Our findings can provide insight for more precise treatment of this drug resistance and cancer treatment crosstalk.

## 1. Introduction

Mucosal melanoma (MM) is a rare and aggressive melanoma subtype that arises in the mucous membranes lining various internal body cavities such as the nasal passages, oral cavity, gastrointestinal tract, and genitourinary tract. Distinct from cutaneous melanoma, MM accounts for only about 1% of all melanomas and is not associated with cutaneous exposure to ultraviolet (UV) light as a risk factor [1]. It is often diagnosed at advanced stages due to anatomical inaccessibility and nonspecific early symptoms, characteristics contributing to its poor prognosis. The five-year survival rate for MM remains below 20%, compared to 90% for cutaneous melanoma [2,3]. This underscores the need for improved diagnostic and therapeutic strategies. The rare incidence and limited sources of human MM cell lines and tissues for research have hampered progress with respect to developing treatments. Canine MM shares conspicuous parallels with human MM [4,5,6], and investigations of naturally occurring MM in pet dogs can serve as an ideal comparative and translational surrogate for human MM [5,7]. Clinically, canine MM mirrors human MM’s invasive growth, frequent metastasis (e.g., to lymph nodes and lungs), poor therapeutic response, and high recurrence rate despite surgery, radiation therapy, or both [8]. Expediently, the spontaneous occurrence of canine MM in an immunocompetent host, with its relative greater incidence in dogs than in humans (up to 100,000 canine diagnoses annually), offers a naturally occurring patient surrogate to develop and refine therapies for both canine and human MM, bridging preclinical and clinical gaps [9,10,11,12].

In contrast to human cutaneous melanoma, there is a lack of recurrent dominant driver mutations to guide the selection of a prevailing targeted therapy for both human and canine MM [13,14,15]. In human MM, 20–40% cases harbor c-KIT mutations [13,16]; however, only approximately 15–21% of these cases include activating mutations in c-KIT (L576P, K642E) [16]. The therapeutic response to c-Kit inhibitors is varied and less than ideal [17]. Similarly, c-Kit mutations are rare in canine MM (<10%) [6,18], and c-kit inhibitors have shown limited efficacy in mouse models [19]. Additionally, BRAF mutation is rare (8%), and NRAS mutations (10–25%) vary in frequency [13,20,21]. Generally, human and canine MM both feature a low overall mutational burden (human: 2–5 m/Mb; canine: 1–4 m/Mb), in contrast to human cutaneous melanoma (10–20 mutations/Mb) [14,22]. Nevertheless, targeting signaling pathways activated in canine and human MM, such as Ras/MAPK and PI3K/AKT/mTOR, remain a viable option due to their frequent activation and vital role in supporting tumor growth [4,23]. Targeting Ras/MAPK and/or PI3K/AKT in canine MM has been shown to inhibit specific target signal transduction while inducing cell death and cell cycle arrest and suppressing tumor growth and metastasis in mouse xenograft models [24].

The Ras/ERK pathway is a pivotal mitogenic cascade that drives cell proliferation by increasing cyclin D expression, a critical regulator of the G_1_-to-S-phase cell cycle transition via CDK4/6 activation [25]. The extracellular signal-regulated kinase (ERK), a key component of Ras signaling, is sequentially activated by Ras and mitogen-activated protein kinase kinase (MEK), which leads to enhanced cyclin D1 (*CCND1*) transcription by phosphorylating transcription factors such as c-Myc and AP-1, thereby transducing extracellular signals into cell cycle progression [26]. Cyclin D1 expression, augmented in several human cancer types, has been found in over 60% of human and canine MM cases [27,28]. Cyclin D1 gene amplification has been associated with the expression of multidrug resistance protein 1 (MDR1) and unfavorable prognosis in multiple myeloma [29]. In solid tumors, cyclin D1 overexpression can be an indicator of tamoxifen resistance and shortened progression-free survival in breast cancer patients [30]. Suppression of cyclin D1 is a consequence of MEK inhibition through allosteric binding by small-molecule inhibitors such as trametinib, which prevents MEK phosphorylation and subsequent activation of ERK1/2 [31,32,33]. Reduced cyclin D1 expression can reverse malignant phenotype and slow tumor growth and increase chemosensitivity [34,35]. Trametinib has demonstrated an objective response rate of approximately 22% in BRAF-mutated human cutaneous melanomas as a monotherapy, with a median progression-free survival (PFS) of 4.8 months [36]. Despite initial efficacy, most patients eventually develop trametinib resistance [37,38], which underscores the need for research into resistance mechanisms and countermeasures. The mechanisms of resistance are diverse and complex, and include target-specific alterations like second-site mutations, amplification of the target kinase, and activation of alternative signaling pathways [39]. Additionally, some 10% of melanoma patients with Ras/MAPK activation fail to respond to targeted inhibition (BRAF and MEK) and are considered intrinsically resistant [40].

Counteracting oncology drug resistance requires a deeper understanding of these mechanisms in the context of disease. Such insights will be instrumental in designing better drug regimens to improve patient outcomes. Investigating mechanisms of drug resistance in canine MM, which exhibits high fidelity for human MM, can inform means to circumvent resistance to targeted therapies, both for their intrinsic value for treating spontaneous canine MM and for comparative translational benefit in human MM [41]. Despite trametinib’s effectiveness in reducing cyclin D1 levels, we noted some canine MM cells were able to proceed with cell cycle progression and proliferate under trametinib treatment in this study. Considering the essential function of D-type cyclins in initiating the cell cycle, we investigated the role of cyclin D family members in canine MM with trametinib resistance.

## 2. Materials and Methods

### 2.1. Cell Lines, Cultures, and Cell Fate Assays

MM cell lines were originally derived ex vivo from canine mucosal melanoma patients and provided by Dr. Michael Kent at University of California, Davis (UCDK9M1 (M1), UCDK9M2 (M2), UCDK9M3 (M3), UCDK9M4 (M4), UCDK9M5 (M5)), and Drs. Jared Fowles and Dan Gustafson of Colorado State University, Fort Collins (Jones) [42,43]. Cells were cultured in DMEM with 10% FBS in 5% CO_2_ at 37 °C. All cell lines were routinely tested for mycoplasma contamination.

M5 cells overexpressing cyclin D2 (M5D2) were generated by co-transfecting M5 cells with XLone-Puro *CCND2*-T2A-luciferase-P2A-eGFP, a gift from Xiaoping Bao Lab, Purdue University (Addgene plasmid # 179843), and Super PiggyBac transposase expression vector (System Biosciences, Palo Alto, CA, USA, PB210PA-1) at a 1:2.5 ratio using jetPRIME transfection reagent (PolyPlus, Illkirch, France). The transfected cells were cultured under puromycin (2.5 µg/mL) selection for two weeks. To induce cyclin D2 expression, 0.1 µg/mL doxycycline (Sigma-Aldrich, Inc., St. Louis, MO, USA) was added to the culture medium one day before experiments.

The enhanced trametinib-resistant M1 (M1T^R^) and M2 (M2T^R^) cells were generated by culturing cells in increasing concentrations of trametinib from 20 nM to 2.0 µM over a period of 2–4 weeks. The M1T^R^ and M2T^R^ cells were then maintained in culture medium containing 2 µM trametinib. Cell titers were determined by MTS assay using CellTiter 96^®^ AQueous Non-Radioactive Cell Proliferation Assay (Promega, Madison, WI, USA) following the manufacturer’s protocol. A total of 3 × 10^3^ cells were seeded in each well of 96-well plates. At the end of treatment, the MTS solution was added to the cells at a 1:5 ratio and incubated for 1.5 h. A microplate reader (SPARK, TECAN, Männedorf, Switzerland) was used to measure the OD at 490 nm.

Cell death was evaluated using Incucyte^®^ Cytotox Dyes and Incucyte^®^ Annexin V Dyes (Sartorius, Göttingen, Germany). A total of 0.5–1 × 10^4^ cells were seeded in each well of 24-well plates 24 h prior to incubation with the drugs and the dyes for the duration of the experiments. Cytotox Dye was used at a final concentration of 250 nM. Annexin V dye was diluted at 1:200 in culture medium. Cell images were acquired every 2–3 h using Incucyte S3 with 10× objectives (Sartorius). Image analyses, confluency, cytotoxicity, and Annexin V labeling were quantified using Incucyte software (v2023A). Each treatment was conducted in triplicate, and a minimum of 10 images were recorded for each well.

### 2.2. Western Blotting

Cell lysates of cultured cell lines were generated by lysing cells in cell lysis buffer (Cell Signaling Technology, Danvers, MA, USA) for 20 min on ice. Cell lysates were cleared by centrifugation at 18,000× *g* for 10 min. The cleared cell lysates were separated on 4–20% Tris-glycine gels (Thermo Fisher Scientific, Inc., Waltham, MA, USA) and transferred onto PVDF membranes (Bio-Rad, Hercules, CA, USA). Membranes were probed with primary antibodies (Supplemental Table S1), followed by respective horseradish peroxidase (HRP)-conjugated secondary antibodies (Jackson ImmunoResearch Laboratories, Inc., West Grove, PA, USA). Immunoreactive bands were detected using chemiluminescence and a GE Amersham Imager 600 (Cytiva, Amersham, UK). The intensities of the signals were quantified using ImageJ (V1.54p). Details of our additional methods, including original blots and relative concentrations, are contained in the Supplementary Materials.

### 2.3. Cell Cycle Flow Cytometry

To analyze the cell cycle, cells were plated one day before treatment application. Briefly, 1 × 10^6^ cells were then washed with PBS at a designated time and fixed with 70% ethanol. RNA was removed by adding 100 units of RNase A (Sigma, St. Louis, MO, USA), and DNA was stained with 50 µg/mL propidium iodide. The cell cycle was analyzed using LSRFortessa (BD Biosciences, San Jose, CA, USA) and ModFit LT 5.0 software.

### 2.4. siRNA Knockdown

For siRNA knockdown (KD) experiments, the cells were plated one day prior to transfection and transfected with 30 nM siRNA targeting *CCND1* or *CCND2* or control siRNA (siGENOME Non-Targeting Control siRNAs, 6505, Dharmacon, Lafayette, CO, USA) using DharmaFECT 1 transfection reagents (Dharmacon). After 24 h incubation, the siRNA was removed, and fresh medium containing trametinib was added to the cells for 24–48 h. Multiple siRNA constructs were tested for each gene (Supplemental Table S1) and showed comparable efficiency (Supplemental Figure S1). A mixture of equal quantities of siRNAs were used in the subsequent experiments.

### 2.5. RNA Extraction and Quantitative RT-PCR Analyses

Total RNA was isolated using RNeasy Plus Kits (Qiagen, Hilden, Germany) following the manufacturer’s manual. RNA quantity and quality were assessed by spectrophotometry (NanoDrop ONE, Thermo Fisher Scientific, Inc., Wilmington, DE, USA). Total RNA was reverse-transcribed using the High-Capacity cDNA Reverse Transcription Kit (Applied Biosystems, Foster City, CA, USA, Cat. # 4368814) according to the manufacturer’s protocol. Quantitative real time PCR (qRT-PCR) analysis was performed using AzuraView™Green Fast qPCR Blue Mix (Azura Genomics, Raynham, MA, USA, Cat. # AZ-2350) and the BIO-RAD CFX connect real-time system (Bio Rad Laboratories, Hercules, CA, USA). Gene expression was normalized to the expression of the housekeeping gene, β-actin. Primer sequences are listed in Supplemental Table S1. Each treatment was conducted in triplicate, and each experiment was repeated at least twice.

### 2.6. EdU Incorporation

EdU incorporation was performed following the siRNA and/or drug treatment using the Click-iT imaging kit (Thermo Fisher Scientific, Inc.). Briefly, cells were pulsed with 10 µM EdU for 1.5 h prior to the fixation, followed by labeling of incorporated EdU according to the manufacturer’s protocol. Cyclin D1 or cyclin D2 immunofluorescent labeling was subsequently performed. Briefly, following EdU labeling, cells were incubated with primary antibodies against cyclin D1 or cyclin D2, Alexa-594 conjugated goat anti-rabbit IgG secondary antibody (1:200 dilution) (Thermo Fisher Scientific, Inc.), and DAPI (1:1000 dilution) (Thermo Fisher Scientific, Inc.) for nuclear counterstaining. Images were captured using the Zeiss Imager M2 device and Zeiss Zen software (version 3.7, Zeiss, Oberkochen, Germany). The percentages of EdU-positive cells were calculated by the number of EdU-positive cells/total (DAPI) cells in each image. Images of ten to twenty-five random fields were taken for each treatment group.

### 2.7. Colony Formation Assay

Cells were seeded in 24-well plates at a density of 2 × 10^3^ cells per well and cultured with trametinib for 1 week. Following the incubation period, the cells were fixed with 20% methanol, and 0.5% crystal violet solution was used to stain and visualize the colonies. Images were captured using Leica M165 FC stereo microscope (Leica Microsystems, Inc., Wetzlar, Germany). Control cells were treated with an equivalent volume of DMSO.

### 2.8. Reagents

Antibodies against p-AKT (Ser473), p-S6 (Ser235/236), p-ERK 1/2, and cyclin D2 were obtained from Cell Signaling Technologies (Danvers, MA, USA). Anti-cyclin D1 and anti-cyclin D2 were obtained from Abcam (Cambridge, MA, USA). Anti-beta-actin was obtained from Sigma-Aldrich (St. Louis, MO, USA). Detailed information is listed in Supplemental Table S1.

Sapanisertib, cobimetinib, selumetinib, and binimetinib were purchased from MedChem Express (Monmouth Junction, NJ, USA). Trametinib was purchased from ChemieTek (Indianapolis, IN, USA). All drugs were dissolved in DMSO to make a stock solution of 10 mM. When treating cells, the drugs were diluted in DMSO to 1000× the designated concentration and added to cell culture medium at 1:1000 (*v*:*v*) dilution. Control cells were treated with an equivalent volume of DMSO.

### 2.9. Statistical Analysis

Statistical analyses were conducted using GraphPad Prism version 10.3.1 or Microsoft Excel. A two-sample unequal variance *t*-test (Welch’s test) was performed to compare two groups. *p* values < 0.05 were considered statistically significant.

## 3. Results

### 3.1. Differential Intrinsic Resistance to Trametinib in a Series of MM Cell Lines

**Figure 1:**
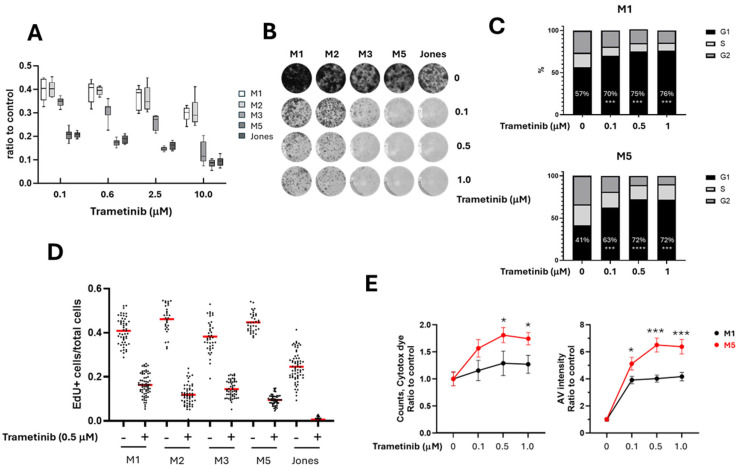
A series of canine MM cell lines exhibited differing sensitivities to acute trametinib exposure. (**A**) Cells were treated with serial concentrations of trametinib for 72 h, and cell viability titers were determined by MTS assay. (**B**) Colony formation assays of canine MM cells treated with trametinib indicate outcomes that parallel viability relationships among cell line series. For a version with an enlarged image resolution, see the Supplemental Methods. (**C**) Trametinib induced G1 cell cycle arrest in both trametinib-resistant (M1) and -sensitive (M5) cells. Cell cycle analyses were performed on M1 and M5 cells in triplicate, with these cells being treated with trametinib for 24 h. (**D**) Cells surviving trametinib treatment retain proliferative capacity. Ratios of cells expressing EdU incorporation to total cells for five MM cell lines, both with and without the addition of trametinib. Red lines represent the mean values. (**E**) Trametinib-sensitive M5 cells (red line) exhibited higher rate of cell death compared to trametinib-resistant M1 cells (black line). M1 and M5 cell were treated with trametinib, and the incidence of cell death was evaluated up to 72 h using Incucyte Cytotox Dye and Annexin V (AV) labeling assays. The average labeling of 10–15 fields of each treatment at 72 h is illustrated to represent the degree of cell death at different trametinib concentrations, referenced to nontreated control conditions. (* *p* < 0.05, *** *p* < 0.001, and **** *p* < 0.0001).

Five canine mucosal melanoma (MM) cell lines were treated with trametinib over a range of concentrations, and cell titers were determined by MTS assays after 72 h. The cell titers at the end of 72 h of treatment revealed different levels of trametinib sensitivity (Figure 1A and Supplemental Table S2). M1 and M2 MM cell lines were relatively resistant to trametinib, while M5 and Jones were more sensitive to trametinib at the concentrations tested. The M3 cell line was intermediate in sensitivity. This relative sensitivity among the cell lines was sustained across a range of trametinib concentrations (Figure 1A). Further analyses of this characteristic distinction, reflected by prototypic M1 trametinib-resistant and M5 trametinib-sensitive cell lines, formed a central focus of the investigation. The differential sensitivity of MM cells to trametinib was further reflected in a colony formation assay (Figure 1B). We also assessed the cell cycle at various trametinib concentrations (0.1, 0.5, and 1.0 µM). Representative results obtained for the relatively resistant (M1) and sensitive (M5) MM cells are shown. In contrast to control cultures lacking trametinib, fractions of cells in the G_1_ cell cycle phase were significantly increased, and the G_2_- and S-phase cell populations were decreased when exposed to trametinib (Figure 1C). An EdU incorporation assay indicated that most cell lines retained the ability to proliferate during trametinib treatment, although at a lower rate compared to untreated control cells (Figure 1D). In addition, the levels of cell death in trametinib-treated M1 and M5 cells mirrored their relative inherent sensitivities (Figure 1E).

### 3.2. Cyclin D Expression Profiles in Canine MM Cells

**Figure 2:**
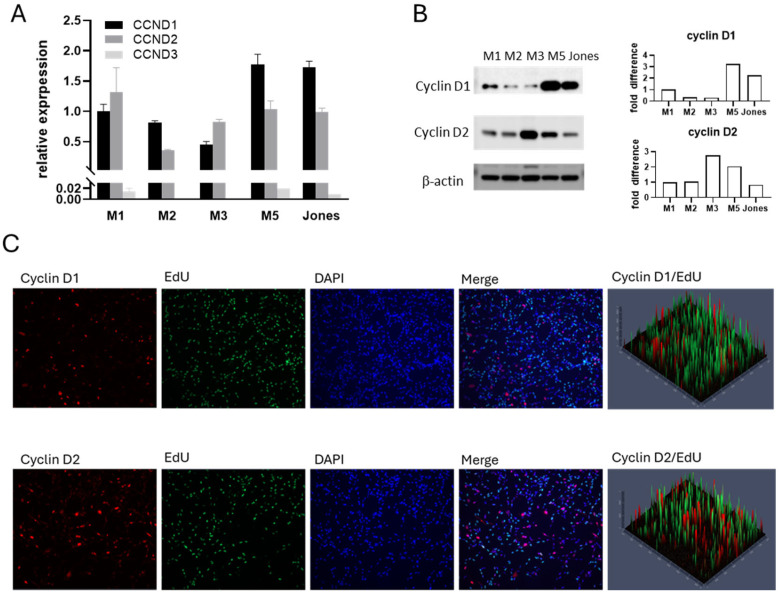
Canine MM cell lines expressed variable levels of cyclin Ds, primarily both cyclin D1 and D2. (**A**) Quantitative PCR detection of cyclins D1, D2, and D3 mRNA levels. (**B**) Cyclin D1 and D2 protein levels were assessed in canine MM cells in the linear growth phase. (**C**) Immunofluorescent labeling of cyclin D1 and cyclin D2 in M5 cells. All MM cell lines examined expressed both cyclin D1 and cyclin D2. Representative immunofluorescent labeling of M5 cells is shown. Fluorescent labeling, Cyclin D1/cyclin D2, red; EdU, green; DAPI/nuclear labeling, blue. The uncropped blots are shown in Supplementary Materials.

Cyclin D plays a pivotal role in cell proliferation by driving progression through the G1 phase and enabling the G1-to-S cell cycle transition. With evidence of differing cell fate under trametinib treatment (Figure 1) and considering that subfamily members of cyclin D have varying roles in driving cell cycle progression, we investigated whether the expression profile of cyclin Ds in canine MM cells was associated with their response to trametinib. All MM cell lines expressed cyclin D1 and cyclin D2 at both transcriptional and protein levels (Figure 2A,B). In contrast, minimal cyclin D3 expression was detected by quantitative PCR (Figure 2A), and cyclin D3 protein was not evident. Nuclear labeling of cyclin D1 and cyclin D2 suggested cells utilize both during the G1 phase (Figure 2C). The non-overlapping pattern of EdU-positive cells (S phase) and nuclear cyclin D positive cells (G1 phase) corresponds to the role of cyclin D in the cell cycle. Endogenous basal levels of cyclin D expression, therefore, did not appear to parallel intrinsic trametinib sensitivity.

### 3.3. Trametinib-Resistance Projected by Compensatory Cyclin D2 Response

**Figure 3:**
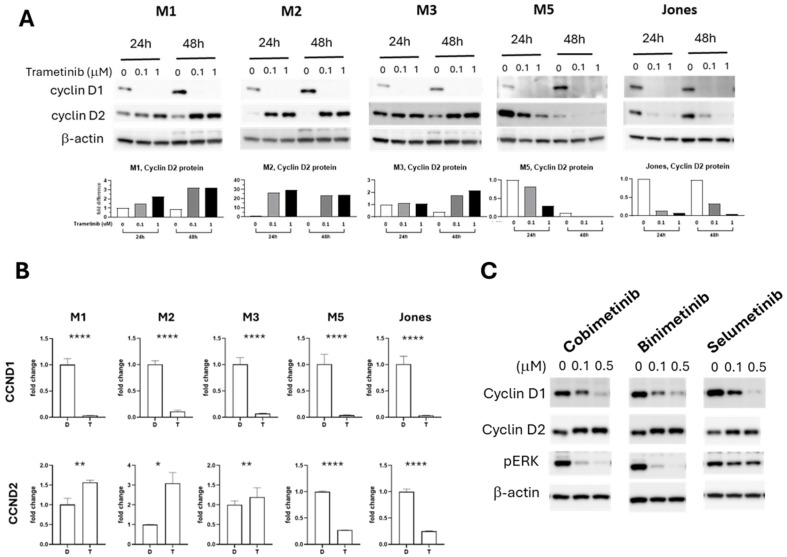
Cyclin D levels were altered in canine MM cell lines upon trametinib exposure and exposure with other MEK inhibitors. (**A**) Cyclin D1 and cyclin D2 immunoblots of canine MM cells treated with 0.1 or 1.0 µM trametinib for 24 and 48 h. Cyclin D2 blot signal intensities were quantitated and normalized with ß-actin. Expression levels are relative to DMSO-treated controls. (**B**) *CCND1* and *CCND2* transcription fold change upon 1.0 µM trametinib (T) treatment for 48 h compared to DMSO (D)-treated control cultures. * *p* < 0.05, ** *p* < 0.01, and **** *p* < 0.0001. (**C**) Other MEK inhibitor treatments elicit similar cyclin D1 and cyclin D2 responses in M1 MM cells. M1 cells were treated with the MEK inhibitors for 48 h at 0.1 and 0.5 µM concentrations. The uncropped blots are shown in Supplementary Materials.

Trametinib reduces the expression of cyclin D1 in canine MM cells [32], as well as cell lines of human non-small-cell lung cancer [44], melanoma [45,46], and colon cancer [47]. Whether trametinib influences the expression of cyclin D2 and cyclin D3 is not well studied. Certain cell types are known to exhibit preferential expression of a particular D-type cyclin, and in the absence of the dominant subtype another D-type cyclin may compensate [48]. Therefore, we subsequently examined how trametinib affected the expression of cyclin Ds in the series of canine melanoma cells. Cells were treated with 0.1 or 1 µM trametinib for 24 and 48 h. The expression of cyclin Ds was documented by Western blot for protein levels and by quantitative PCR for gene transcription (Figure 3A,B). Upon trametinib treatment, cyclin D1 was diminished in all five MM cell lines. The cyclin D2 responses, however, were noteworthy for their corresponding differences among cells across the range of trametinib sensitivities. The cyclin D2 level in the trametinib-sensitive cell lines, M5 and Jones, was decreased, along with cyclin D1. By contrast, relatively resistant MM cells (M1 and M2) upregulated cyclin D2 expression, as cyclin D1 was downregulated. In M3 cells, the intermediate responder to trametinib, a delayed and more muted cyclin D2 response was observed; cyclin D2 remained comparable with or without trametinib treatment at 24 h, while at 48 h, cyclin D2 level was slightly increased (Figure 3A). Cyclin D3 levels were relatively low compared to cyclin D1 and cyclin D2, with or without trametinib treatment, in all five MM cells (Supplemental Figure S2). The reciprocal expression of cyclin D2 upon cyclin D1 reduction during MEK inhibition in MM did not appear to be trametinib-specific. The effects of other small-molecule MEK inhibitors, including cobimetinib, binimetinib, and selumetinib, on cyclins D1 and D2 were examined using the prototypic trametinib-resistant M1 cells. The relative influences on cyclin D1 and D2 expression were similar to that of trametinib (Figure 3C).

The dynamic reciprocal expression of cyclin D1 and cyclin D2 in MM was further tested by knocking down (KD) cyclin D1 with siRNA. The more resistant M1 and M2 cells responded to cyclin D1 KD by increasing the expression of cyclin D2, while intrinsically more sensitive M3, M5, and Jones MM cells showed no substantive changes in cyclin D2 level when cyclin D1 expression was reduced by siRNA (Supplemental Figure S3A). In the absence of trametinib, cyclin D1 KD with siRNA by itself did not significantly alter M1 or M5 proliferative capacity (Supplemental Figure S3B), indicating that the presence of cyclin D2 was sufficient to sustain cell proliferation. Reduced proliferation was observed as a response to trametinib, although when combined with cyclin D1 KD in this series of experiments using siRNA, no further reduction in proliferation was observed (Supplemental Figure S3B).

### 3.4. Inhibition of Compensatory Cyclin D2 Expression Conferred Sensitivity in Trametinib-Resistant MM

**Figure 4:**
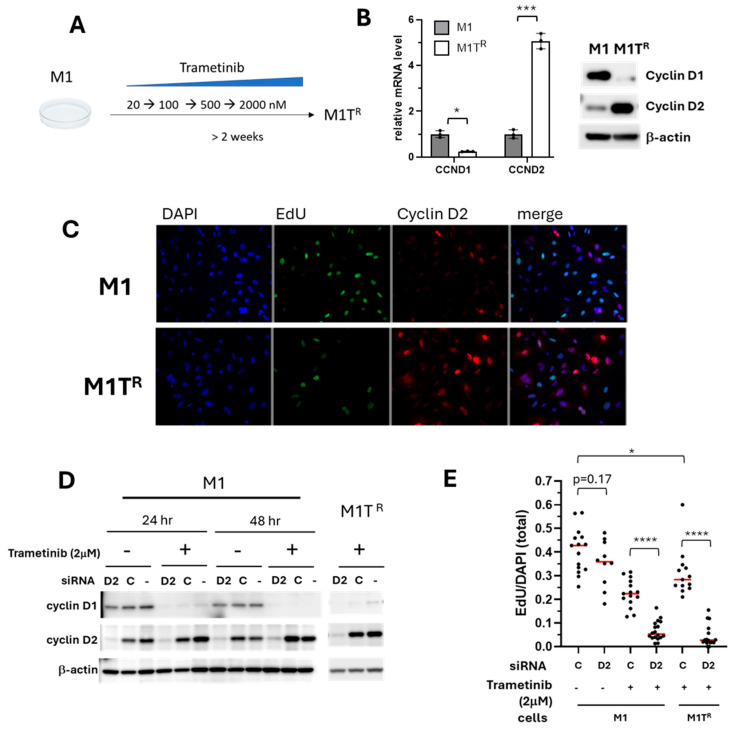
Cyclin D2 expression is critical for the proliferative capacity of trametinib-resistant M1 (M1T^R^) cells. (**A**) M1T^R^ cells were generated by adaptive exposure of parental M1 cells to increasing concentrations of trametinib for 2–4 weeks to establish the enhanced resistance phenotype. M1T^R^ cells subsequently tolerated maintenance in culture medium containing 2 µM trametinib. (**B**) Cyclin D1 was decreased in M1T^R^ cells at transcription and protein levels, while cyclin D2 was increased compared to parental M1 cells. (**C**) Immunofluorescent labeling of cyclin D2 demonstrated an increased cyclin D2 expression in M1T^R^ cells. The non-overlapping nuclear localization of cyclin D2 and EdU labeling corresponded to its function in cell cycle. Fluorescent labeling, cyclin D2, red; EdU, green; DAPI/nuclear labeling, blue. (**D**) Cyclin D2 was reduced in M1 and M1T^R^ cells by siRNA KD. M1 and M1T^R^ cells were transfected with *CCND2*-specific siRNA (D2), control siRNA (C), or no siRNA (−) for 24 h, followed by 2 µM trametinib treatment for 24 h, representing the maintenance concentration for M1T^R^ cell culture. The KD efficacy was determined by Western blot (Supplemental Figure S1B). (**E**) The impact of cyclin D2 KD on cell proliferation in M1 and M1T^R^ maintained in trametinib was assessed by EdU labeling. Cell proliferation was significantly compromised by siRNA KD of cyclin D2 in trametinib-treated M1 and M1T^R^ cells. (* *p* < 0.05, *** *p* < 0.001, and **** *p* < 0.0001). Red lines represent the mean values.

Enhanced trametinib-resistant M1 (M1T^R^) and M2 (M2T^R^) cells were generated to further investigate the role of the reciprocal expression of cyclin D1 and cyclin D2 in MM trametinib resistance. The more resistant M1T^R^ and M2T^R^ cells were adapted to trametinib by culturing the respective parental cell lines in increasing concentrations of trametinib over 2–4 weeks, reaching tolerance for 2 µM concentration (Figure 4A). Cyclin D1 was decreased and cyclin D2 was increased in both M1T^R^ and M2T^R^ cells, relative to the respective parental lines (Figure 4B–D and Figure S4), demonstrating maintenance of the cyclin D expression pattern that was exhibited during short-term trametinib treatment (Figure 3).

Further examination of the potential role that compensatory cyclin D2 expression played in MM cells surviving under trametinib treatment was undertaken using siRNA to KD cyclin D2 expression. Under growth conditions lacking trametinib, cyclin D2 KD did not significantly reduce cell proliferation compared to cells transfected with control siRNA (*p* = 0.17), results that were similar to cyclin D1 KD alone (Figure 4E, and Figures S3 and S4B). This is consistent with our finding that canine MM cell lines appear to have the capacity to use cyclin D1 and cyclin D2 interchangeably under some circumstances (Figure 2C). Cyclin D2 KD in trametinib-treated cells, however, further reduced the cell proliferative capacity compared to M1 and M1T^R^ cells transfected with control siRNA (*p* < 0.0001) (Figure 4E). Similar results were observed for trametinib-treated M2 and for the conditionally adapted M2T^R^ cells (Supplemental Figure S4B). Reduced proliferative capacity in response to cyclin D2 KD provided evidence that reciprocal cyclin D2 upregulation played a necessary compensatory role when cyclin D1 was downregulated by trametinib.

### 3.5. Induced Overexpression of Cyclin D2 in MM Confers Greater Trametinib Resistance

To further investigate the essentiality of compensatory cyclin D2 upregulation in the observed trametinib resistance among MM cells, the trametinib-sensitive M5 cell line was engineered to express a Tet-on inducible cyclin D2 (M5D2). The expressed recombinant human cyclin D2 shares 95% protein sequence homology with canine cyclin D2 (Supplemental Figure S5A), with molecular weights of 33 kDa and 32.6 kDa, respectively. The recombinant cyclin D2 protein is slightly larger due to the presence of a residual T2A peptide at the carboxyl-terminus (Supplemental Figure S5B,C).

**Figure 5:**
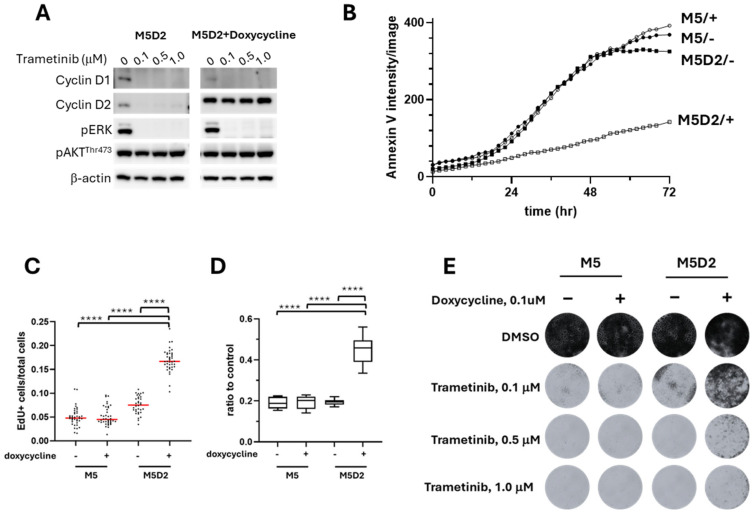
Induced cyclin D2 overexpression in M5 canine MM cells (M5D2) conferred greater resistance compared to the trametinib-sensitive phenotype of parental M5 cells. (**A**) The expression of cyclin D1, cyclin D2, p-ERK, and p-AKT in M5D2 cells with or without induced cyclin D2 expression. M5D2 with or without doxycycline-induced recombinant cyclin D2 were treated with trametinib at 0.1, 0.5, or 1.0 µM for 48 h. (**B**) Annexin V-red dye was added to cells along with 0.5 μM trametinib to monitor the cell death. The intensity of red fluorescence was measured every 2 h for 72 h. Values for M5D2/+ were significantly different from other groups after 24 h (*p* < 0.01). (**C**) EdU incorporation was performed 48 h after 0.5 μM trametinib treatment. The ratios of EdU-positive cells to the total cells were used to quantify the proliferative activities. Each dot represents one image, and each treatment was performed in triplicate. **** *p* < 0.0001. Red lines represent the mean values. (**D**) MTS assay was performed to measure the cell titer 72 h after 0.5 μM trametinib treatment. **** *p* < 0.0001. (**E**) Colony formation assay revealed an increased cell survival in M5 cells exposed to trametinib when cyclin D2 overexpression was induced. For a version with an enlarged image resolution, see Supplemental Methods.

Induced recombinant cyclin D2 expression in M5D2 cells was associated with diminished expression of endogenous cyclin D1 (Supplemental Figure S5B). Similarly to the parental M5 cells (Figure 3A), trametinib treatment reduced the expression of endogenous cyclins D1 and D2 in M5D2 cells but had no apparent effect on induced recombinant cyclin D2 as assessed by Western blot (Figure 5A). This result suggested that the decreased cyclin D1 and D2 in trametinib-treated M5 and M5D2 cells was likely due to reduced transcription/translation of endogenous cyclins rather than increased protein degradation. Under trametinib treatment, M5D2 cells with induced recombinant cyclin D2 exhibited significantly less apoptotic cell death (Figure 5B). Resistance to cell death due to induced expression of cyclin D2 (M5D2 + doxycycline) was accompanied by superior cell proliferation (Figure 5C) and, as a result, greater cell titers (Figure 5D and Figure S6) with more proficiently formed colonies under trametinib treatment (Figure 5E). The results indicated that induced overexpression of cyclin D2 compensated for the loss of cyclin D1 and promoted trametinib resistance in otherwise treatment-sensitive M5 MM cells, which lacked an intrinsic capacity to upregulate endogenous cyclin D2 in response to trametinib (Figure 3A).

### 3.6. PI3K/AKT/mTOR Signal Transduction Pathway Inhibition SuppressedTrametinib-Stimulated Compensatory Cyclin D2 Expression

**Figure 6:**
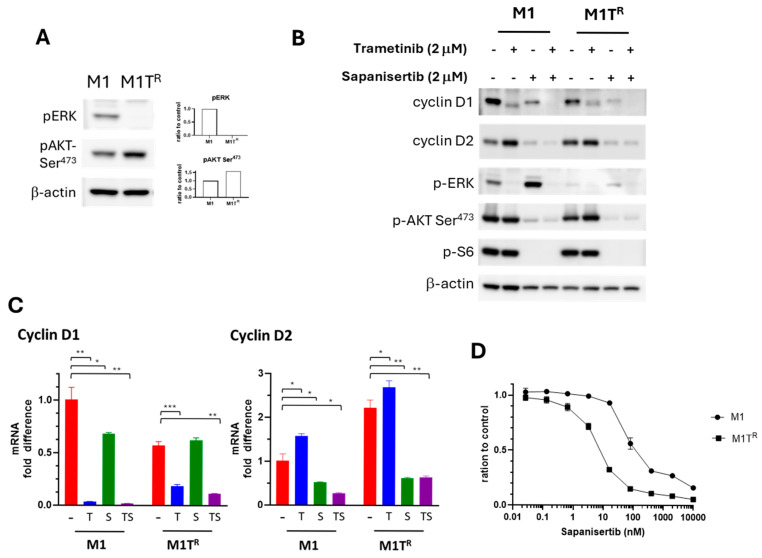
Sapanisertib, an mTORC1/2 inhibitor, countered increased cyclin D2 expression induced by trametinib and affected survival of M1 and M1T^R^ MM. (**A**) M1T^R^ cells with adapted trametinib resistance exhibited increased levels of pAKT concurrent with pERK diminution. (**B**) Sapanisertib alone, and in combination with trametinib, decreased levels of cyclin D1 and cyclin D2 and inhibited AKT and S6 phosphorylation. (**C**) mTORC1/2 inhibitor sapanisertib (S) inhibited cyclin D2 transcription in M1T^R^ cells (shown compared to M1T^R^ treated with trametinib alone (T), compared to when combined with trametinib (TS), and compared to control (−)). Results are similar for parental M1 cells. Sapanisertib is less effective in reducing *CCND1* transcription compared to T. Cells were treated with 2 μM trametinib and/or sapanisertib for 48 h. (* *p* < 0.05, ** *p* < 0.01, and *** *p* < 0.001). (**D**) Significantly decreased survival of M1T^R^ cells exposed to sapanisertib compared to parental M1 cell line (*p* = 0.0066). The uncropped blots are shown in Supplementary Materials.

We previously documented that canine MMs treated with trametinib are capable of reciprocal activation of PI3K/AKT/mTOR pathway [49]. In the current study, reduced ERK phosphorylation in trametinib-resistant M1T^R^ cells was accompanied by elevated AKT activation, indicating the capacity for reciprocal PI3K/Akt/mTOR signaling (Figure 6A). M1T^R^ cell reliance on PI3K/AKT pathway for survival was tested by treating M1 and M1T^R^ cells with sapanisertib, a dual mTORC1/2 inhibitor. Sapanisertib treatment reduced pAKT and pS6, inhibiting PI3K/AKT pathway signaling in both M1 and M1T^R^ cells (Figure 6B). Sapanisertib treatment led to a reduction in cyclin D1 expression (Figure 6B,C). In the case of cyclin D2, sapanisertib not only suppressed basal cyclin D2 expression in M1 cells but also inhibited the trametinib-induced upregulation of cyclin D2 in M1T^R^ and M1 cells treated with trametinib (Figure 6B,C). Notably, M1T^R^ cells were more susceptible to sapanisertib treatment than parental M1 cells (Figure 6D), further demonstrating the dependency of the conditionally adapted trametinib-resistant M1T^R^ cells on the PI3K/AKT signaling pathway for survival.

## 4. Discussion

Despite initial treatment effectiveness, resistance to cancer therapy develops all too frequently, permitting further tumor growth and presenting a significant hurdle in achieving cancer disease remission. A variety of mutational and non-mutational events as well as changing microenvironmental factors during targeted therapy can result in resistance mechanisms for bypassing oncogenic signaling inhibitors [40]. Principal oncogenic signal transduction in melanoma often engages Ras/MAPK activation and can co-involve, or recruit, dysregulated PI3K/AKT/mTOR signaling that can serve as a source of resistance to MAPK inhibition, including in the case of human and canine MM [4,23,50]. D-type cyclins function as important sensors for growth factor signaling transduced by both the Ras/MAPK and PI3K/AKT/mTOR pathways [51]. For example, induction of cyclin D1 requires MAPK signal transduction for most cells to initiate and progress in G_1_- to S-phase transitions [46]. When dysregulated, overexpression or otherwise aberrant accumulation of cyclin D1 can be manifest in a variety of solid human cancers, consistent with an oncogenic function [52]. Preservation of cancer cell proliferative capacity is a fundamental mechanism in therapeutic escape.

In the current study, maintenance of proliferative capacity in early resistance to trametinib is linked with compensatory cyclin D expression. Through examination of five canine patient-derived MM cell lines, we noted the existence of MM cell lines that were susceptible to trametinib and those that appeared relatively more intrinsically resistant. Prior to any treatment, all cell lines expressed varying levels of both cyclins D1 and D2. Trametinib reduced cyclin D1 expression across all cell lines, consistent with rational targeting expectations [53]. As cyclin D1 was reduced in these experiments, cyclin D2 upregulation promoted proliferation in the more trametinib-resistant cells (M1 and M2), in contrast to the trametinib-sensitive cells (M5 and Jones) that did not exhibit similar capacity for compensatory cyclin D2 upregulation. This compensatory resistance was reversed by blocking cyclin D2 expression using siRNA and by pharmacologic inhibition of the PI3K/AKT/mTOR pathway. Furthermore, the compensatory cyclin D2 upregulation was exhibited in the more resistant canine MM cell lines exclusively following siRNA KD of cyclin D1. Conversely, increased cell proliferative capacity was observed in trametinib-sensitive cells when exogenous cyclin D2 was introduced. Therefore, compensatory cyclin D2 upregulation in response to MEK inhibitor exposure and the diminution of cyclin D1 in this study appears to be a crucial and reciprocal resistance characteristic for maintaining cell proliferation in MM cells with this capacity. These results provide valuable insights into a mechanism of resistance to trametinib. The valuable insight gained also appears to have translational relevance for human cancer. Although knowledge regarding the roles of D type cyclins in cancer resistance is limited, reciprocal expression of cyclins D1 and D2 was reported in one human melanoma patient during the development of resistance to trametinib [54]. Analogous activated Ras/MAPK pathway-based targeted inhibition using vemurafenib [54], or combination dabrafenib/trametinib [55] therapy in melanoma patients, also resulted in a similar compensatory cyclin D switch.

Among the D-type cyclins, cyclin D1 and cyclin D2 generally exhibit cell-type-specific expression. Cyclin D1 is predominantly found in epithelial and certain mesenchymal cells, whereas cyclin D2 is primarily expressed in myelomonocytic/lymphoid cells and pancreatic beta cells [56,57]. This specificity suggests distinct regulatory mechanisms for each cyclin across different cell types. Yet, despite their preferential tissue distribution, cyclin D1 and cyclin D2 can functionally substitute for one another. Reciprocity of D-type cyclin expression has been observed in various biological contexts, emphasizing their distinct yet compensatory roles in cell cycle regulation [51]. For instance, cyclin D2 can compensate for the loss of cyclin D1 in estrogen-driven proliferation of uterine epithelial cells [48]. In cyclin D1-null mice, cyclin D2 was able to form complexes with CDK4, translocate to the nucleus, and perform typical cyclin D1/CDK4 functions, ensuring normal cell cycle progression [48]. Additionally, cyclin D2 could rescue retinal and mammary gland developmental defects in cyclin D1 knockout mice when it was knocked in to replace cyclin D1 [58]. These findings indicate that although functional differences among D-type cyclins have been identified in distinct tissues, some of their functions do overlap, allowing them to compensate for one another under certain conditions [25].

Cyclin D2 expression and activity has been shown to be influenced by the activation of mTOR, which comprises two distinct multi-protein complexes (mTORC1 and mTORC2) involved in regulating cellular metabolism, growth, and proliferation [59,60]. For example, the mTORC1 impacted cyclin D2 levels in pancreatic beta cells and T cells [59,61]. Controlled increases in AKT activities led to increased mTORC1 activity along with elevated cyclin D2 level, and the increased cyclin D2 was considered to result from enhanced protein translation and increased stability [59]. Further evidence that mTORC1 regulates cyclin D2 expression primarily at the post-transcriptional level, affecting both the synthesis and stability of the cyclin D2 protein, was found through functional mTORC1 disruption in a Raptor-null mouse model, which affected the stability of the cyclin D2/CDK6 complex in T cells [61].

mTORC2, on the other hand, has been shown to affect cyclin D2 expression indirectly. mTORC2 directly phosphorylates AKT at serine 473, a modification necessary for complete AKT activation. This phosphorylation event enhances AKT’s kinase activity, enabling it to phosphorylate and suppress downstream substrates, including Forkhead box O (FOXO) transcription factors (TFs) and glycogen synthase kinase 3 (GSK3) [62]. FOXO TFs are known suppressors of cyclin D2 expression; the phosphorylation of FOXO TFs targets them for degradation, thereby lifting their suppressive effect on cyclin D2 transcription. GSK3β phosphorylates cyclin D2, targeting it for degradation through the ubiquitin–proteasome pathway [63]. Through the activation of AKT and the subsequent inhibition of GSK3β and FOXO TFs, mTORC2 indirectly promotes the expression, stabilization, and accumulation of cyclin D2.

In cancers, cyclin D2 has been associated with tumor enhancer [64,65,66] and suppressor functions [67,68]. Although typically associated with hematologic malignancies, activating/sensitizing *CCND2* gene alterations reportedly occur in up to 23% of testicular cancers [69] and may occur infrequently in some cases of human gastric carcinoma [70]. In its interactions with CDK4/6, cyclin D2 has been shown to play an essential role in cell cycle progression for undifferentiated glial stem cells and in experimental gliomagenesis [66].

Inhibition of ERK activation, according to the current study and previous reports, can lead to reciprocal PI3K/AKT/mTOR crosstalk activation, thereby conferring resistance to MEK inhibition [32,49]. In MM manifesting greater trametinib resistance that mounted AKT activation in the face of MEK inhibition, we also found elevated cyclin D2 expression. Congruent with the known effects of mTOR signaling on cyclin D2 expression, a diminished cyclin D2 (and D1) level occurred, along with reductions in mediators of PI3K/AKT/mTOR pathway signal transduction as a response to sapanisertib treatment, a dual inhibitor of mTORC1 and mTORC2 in this study. Sapanisertib’s inhibitory impact in trametinib-resistant M1T^R^ cells further supports dependency of the conditionally adapted trametinib-resistant M1T^R^ cells on the PI3K/AKT signaling pathway and on cyclin D2 compensation for their survival under trametinib pressure. Therefore, inhibiting cyclin D2 may contribute to the previously noted synergistic effect overcoming the resistance observed, particularly when sapanisertib is combined with MEK inhibition [49]. Two-drug combinations that target MAPK and PI3K/Akt signaling pathways in parallel impart synergistic inhibition of tumor growth and metastasis in mouse models of MM with Ras/MAPK and PI3K/AKT/mTOR activation [32,49]. Additional means for targeting potential cyclin D2 compensation may also prove beneficial. Intriguing avenues for future investigation of transcriptional/translational regulation of the relationship implicated between PI3K/AKT/mTOR signaling and cyclin D2 compensation would logically include a need for cloning the canine *CCND2* gene 5′ promoter region to evaluate binding factors for drug-resistant and sensitive cells.

## 5. Conclusions

The findings of this study substantiated that the compensatory cyclin D2 response played a role in imparting resistance to MEK inhibition by maintaining MM proliferative capacity during trametinib treatment. The results indicate the likelihood that some MM tumors have an intrinsic capacity to mount a cyclin D subtype-related compensatory response to trametinib, while other tumors would be less competent and thereby more susceptible to MEK inhibition. Current research has not sufficiently provided a molecular correlate of the nature of this characteristic reciprocity, and additional studies are needed. Examining the role that compensation among cyclin D subtypes plays in contributing to escape from trametinib inhibition in canine MM may provide further insight for countering resistance driven through analogous network crosstalk mechanisms in this and other cancers.

## Data Availability

The authors confirm that the data supporting the findings of this study are available within the article. Other inquiries from investigators for data availability sup-porting the findings of this study may be directed to the corresponding author.

## Supplementary Materials

The following supporting information can be downloaded at: https://www.mdpi.com/article/10.3390/cancers17142357/s1, Figure S1: siRNA efficacy in vitro; Figure S2: Quantitative PCR of D-type cyclins in treated and untreated cells; Figure S3: MM cell response to cyclin D1 knockdown; Figure S4: Cyclin D2 knockdown with and without treatment in M2 and M2Tr cells; Figure S5: Induced cyclin D2 expression system in M5 MM cells; Figure S6: (A) M5D2 cells treated with trametinib and doxycyclin.avi; (B) M5D2 cells treated with trametinib without cyclin D2 induction.avi; Table S1: Reagents-siRNA, primers and antibodies; Table S2. Multiple two-way comparisons between mucosal melanoma cell line viabilities over a range of trametinib concentrations.

## Abbreviations

The following abbreviations are used in this manuscript:

AV	Annexin V
DAPI	4′,6-diamidino-2-phenylindole
DMSO	Dimethyl Sulfoxide
EdU	5-ethynyl-2′-deoxyuridine
GE	General Electric
HRP	Horseradish Peroxidase
KD	Knockdown
MM	Mucosal Melanoma
M5D2	M5 Cells Overexpressing Cyclin D2
M1T^R^	M1 Cells Conditionally Adapted to Trametinib
M2T^R^	M2 Cells Conditionally Adapted to Trametinib
MTS	3-(4,5-dimethylthiazol-2-yl)-5-(3-carboxymethoxyphenyl)-2-(4-sulfophenyl)-2H-tetrazolium
PBS	Phosphate-Buffered Saline
PVDF	Polyvinylidene Difluoride
