# Supplementary material for: Capacity for Compensatory Cyclin D2 Response Confers Trametinib Resistance in Canine Mucosal Melanoma
Source: Cancers (Basel). 2025 Jul 15;17(14):2357. doi: 10.3390/cancers17142357 (PMC12293520; doi:10.3390/cancers17142357)
Supplement: Supplementary file 1 [file cancers-17-02357-s001.zip › cancers-3701192-WB figures.pdf]

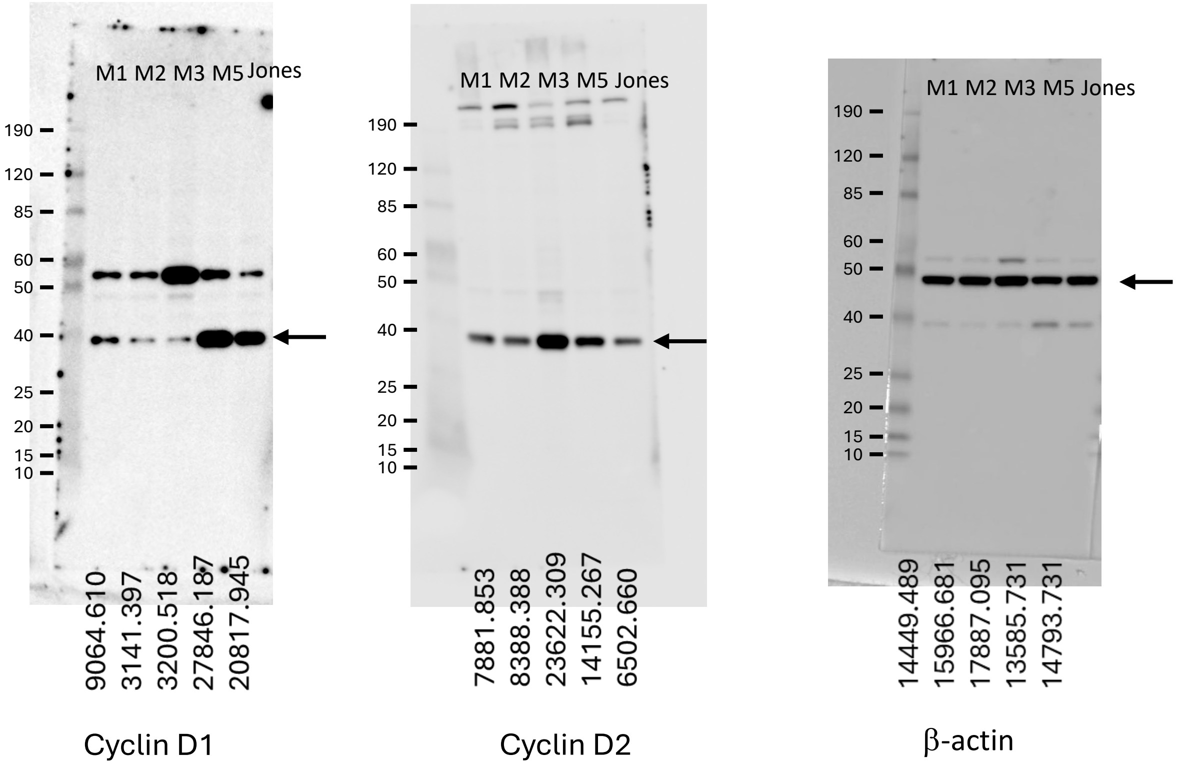
M1 M2 M3 M5 Jones
190
120
85
60
50
40
25
20
15
10
9064.610
3141.397
3200.518
27846.187
20817.945
Cyclin D1
M1 M2 M3 M5 Jones
190
120
85
60
50
40
25
20
15
10
7881.853
8388.388
23622.309
14155.267
6502.660
Cyclin D2
M1 M2 M3 M5 Jones
190
120
85
60
50
40
25
20
15
10
14449.489
15966.681
17887.095
13585.731
14793.731
β-actin

Figure 2B

Figure 3A

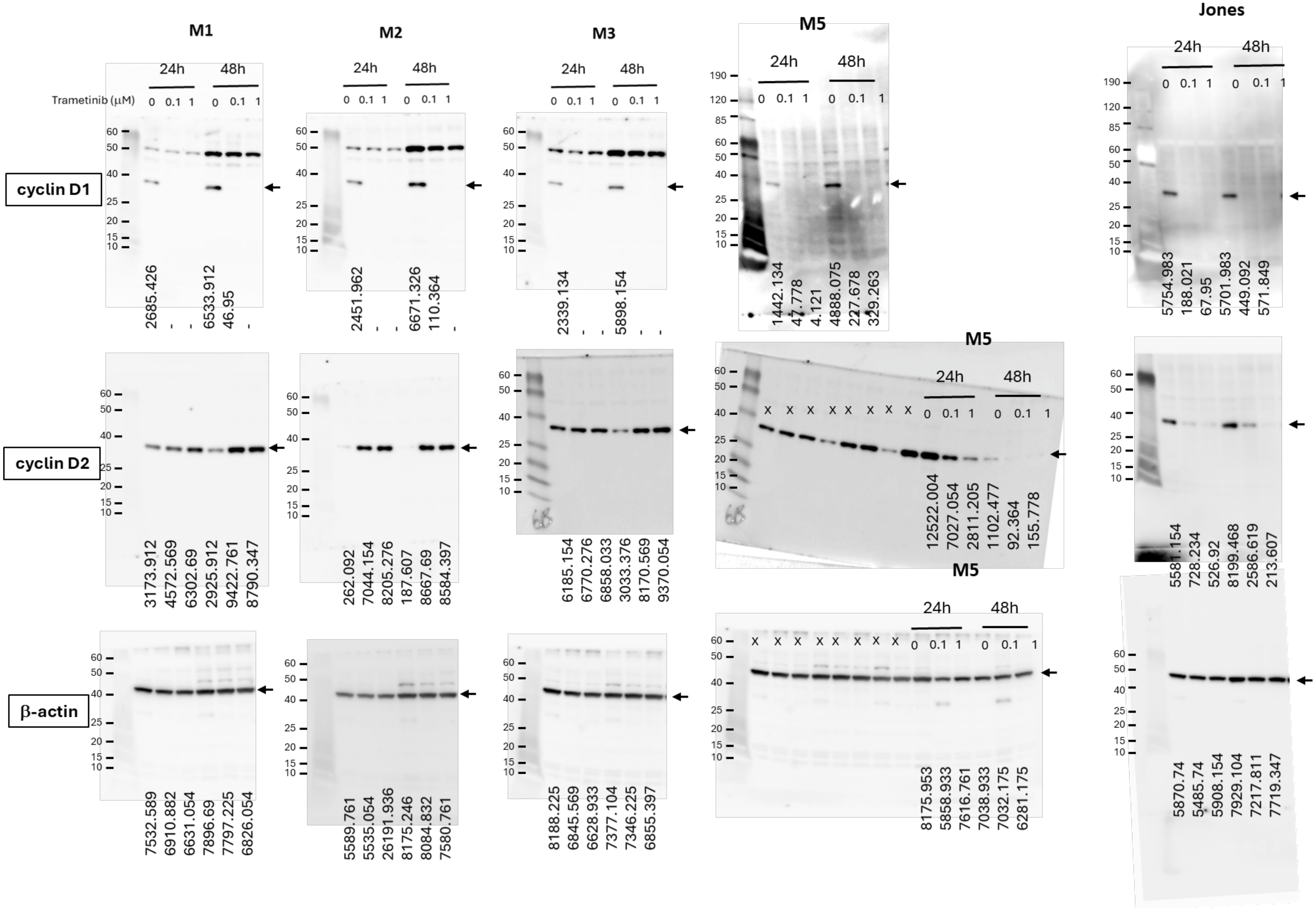

M1
M2
M3
M5
Jones
24h
48h
Trametinib (μM)
0 0.1 1 0 0.1 1
cyclin D1
cyclin D2
β-actin
2685.426
6533.912
46.95
2451.962
6671.326
110.364
2339.134
5898.154
1442.134
47.778
4.121
4888.075
227.678
329.263
5754.983
188.021
67.95
5701.983
449.092
571.849
3173.912
4572.569
6302.69
2925.912
9422.761
8790.347
262.092
7044.154
8205.276
187.607
8667.69
8584.397
6185.154
6770.276
6858.033
3033.376
8170.569
9370.054
12522.004
7027.054
2811.205
1102.477
92.364
155.778
5581.154
728.234
526.92
8199.468
2586.619
213.607
7532.589
6910.882
6631.054
7896.69
7797.225
6826.054
5589.761
5535.054
26191.936
8175.246
8084.832
7580.761
8188.225
6845.569
6628.933
7377.104
7346.225
6855.397
8175.953
5858.933
7616.761
7038.933
7032.175
6281.175
5870.74
5485.74
5908.154
7929.104
7217.811
7719.347

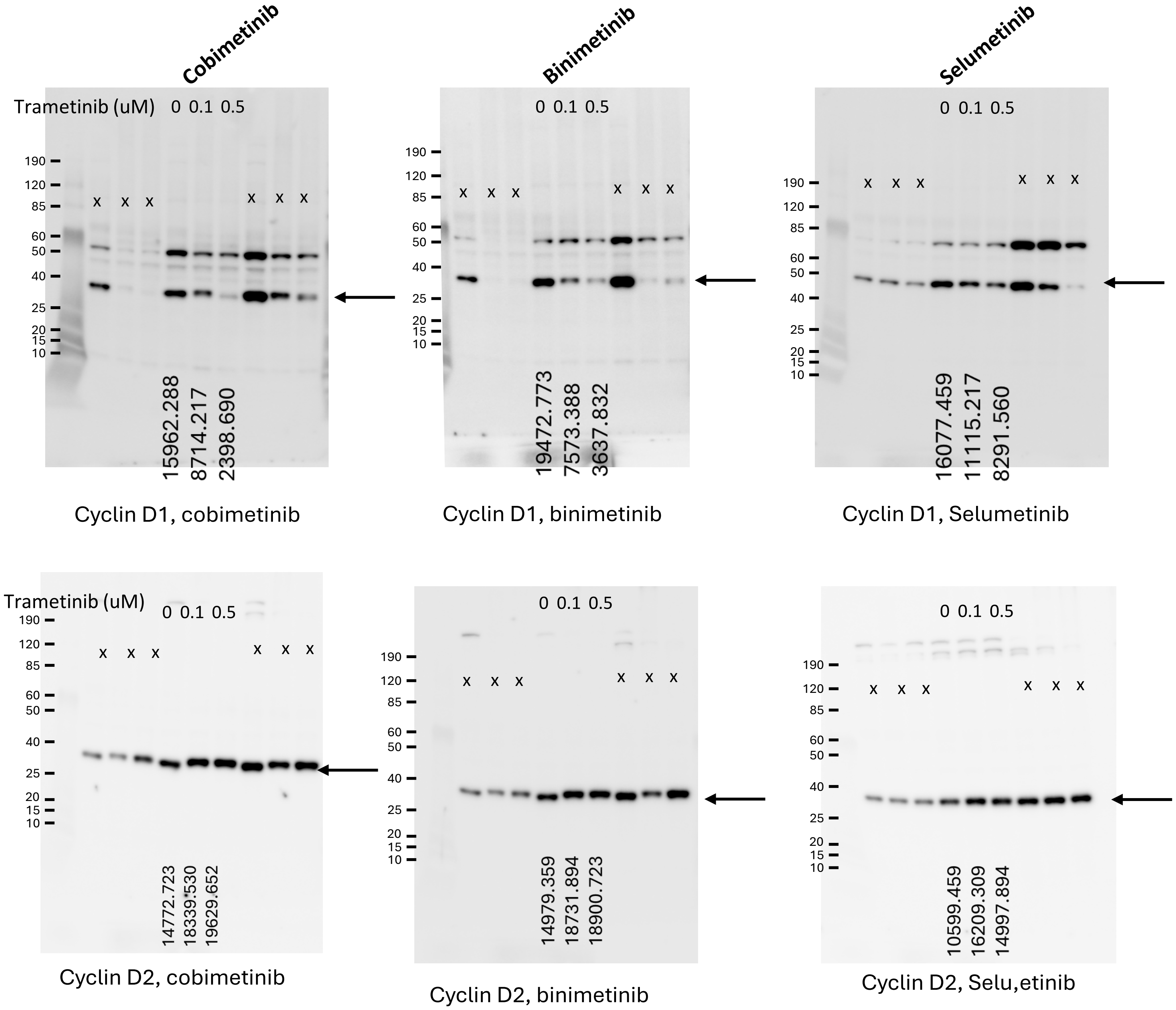

Cobimetinib
Binimetinib
Selumetinib
Trametinib (uM)
0 0.1 0.5
190
120
85
60
50
40
25
20
15
10
x x x x x x
15962.288
8714.217
2398.690
Cyclin D1, cobimetinib
19472.773
7573.388
3637.832
Cyclin D1, binimetinib
16077.459
11115.217
8291.560
Cyclin D1, Selumetinib
14772.723
18339.530
19629.652
Cyclin D2, cobimetinib
14979.359
18731.894
18900.723
Cyclin D2, binimetinib
10599.459
16209.309
14997.894
Cyclin D2, Selu,etinib

Figure 3C

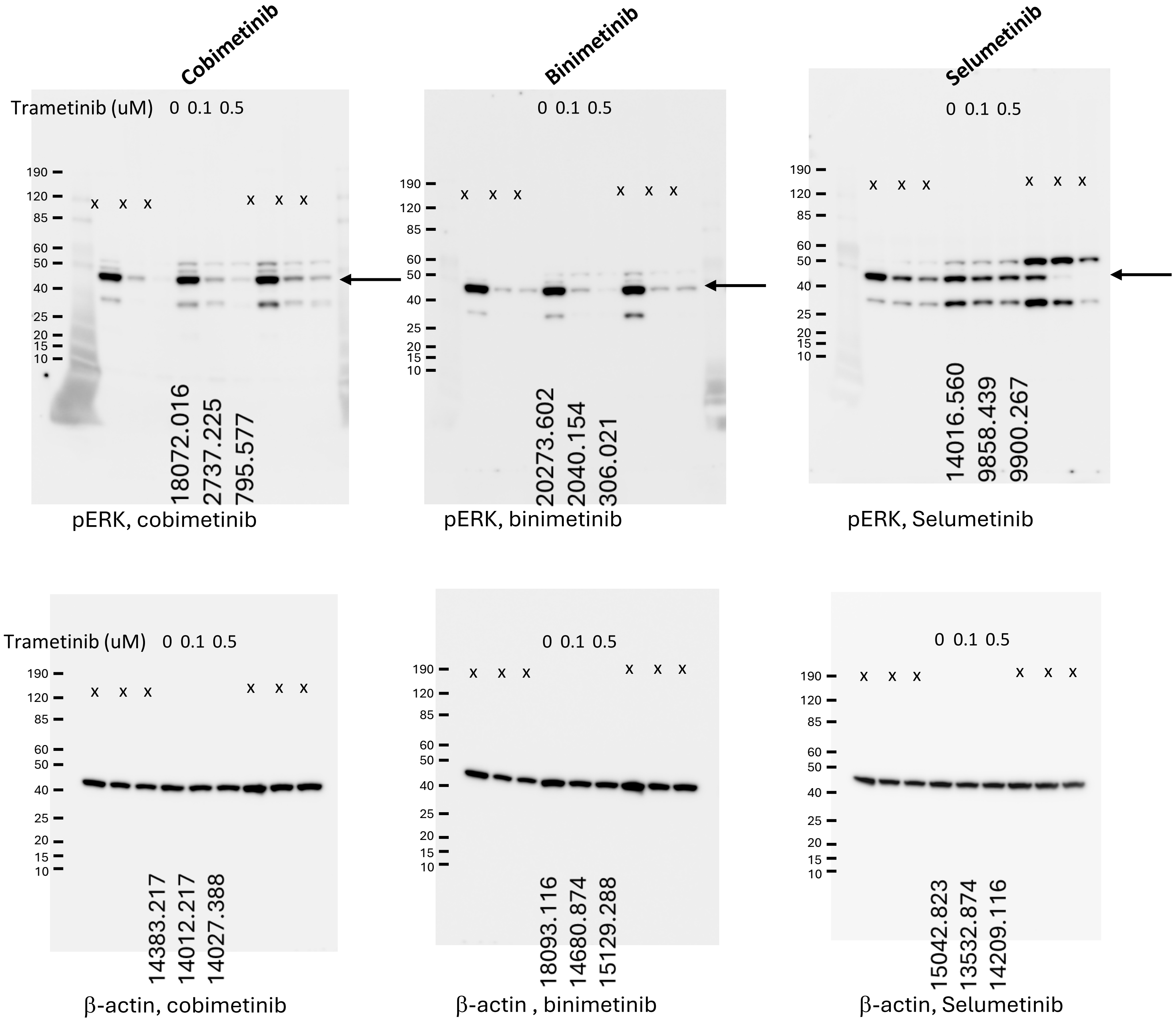
Cobimetinib
Binimetinib
Selumetinib
Trametinib (uM) 0 0.1 0.5
0 0.1 0.5
0 0.1 0.5
190
120
85
60
50
40
25
20
15
10
x x x
x x x
18072.016
2737.225
795.577
20273.602
2040.154
306.021
14016.560
9858.439
9900.267
pERK, cobimetinib
pERK, binimetinib
pERK, Selumetinib
Trametinib (uM) 0 0.1 0.5
0 0.1 0.5
0 0.1 0.5
14383.217
14012.217
14027.388
18093.116
14680.874
15129.288
15042.823
13532.874
14209.116
β-actin, cobimetinib
β-actin , binimetinib
β-actin, Selumetinib

Figure 3C

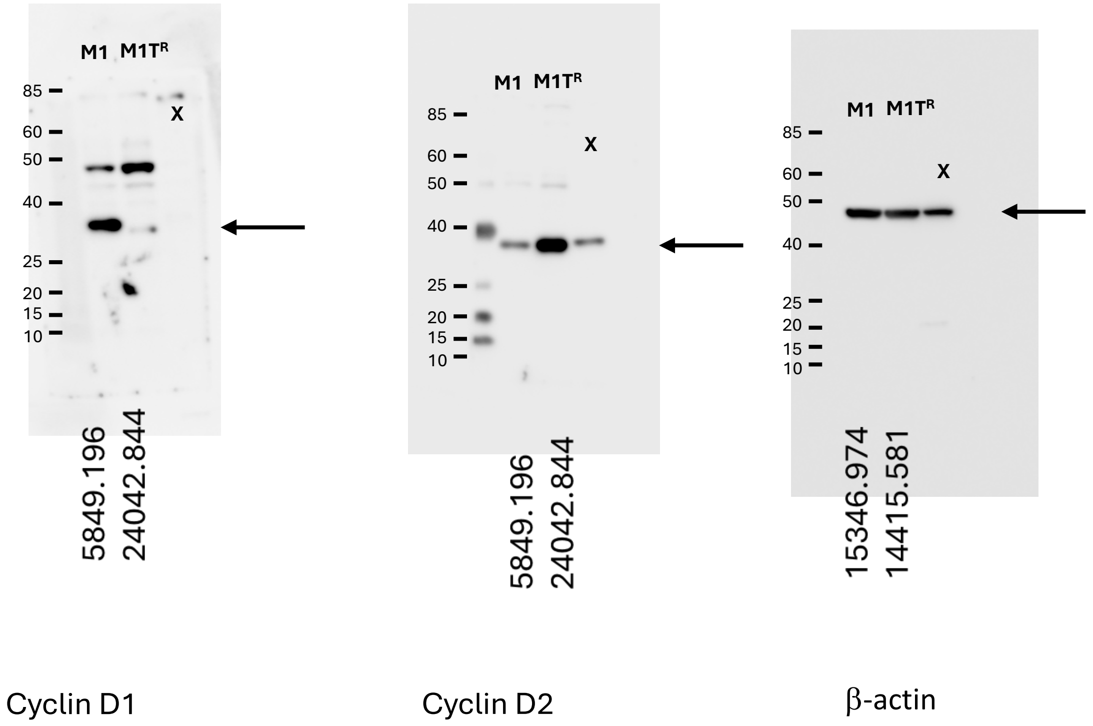

M1 M1T^R
85
60
50
40
25
20
15
10
X
5849.196
24042.844
15346.974
14415.581
Cyclin D1
Cyclin D2
β-actin

Figure 4B

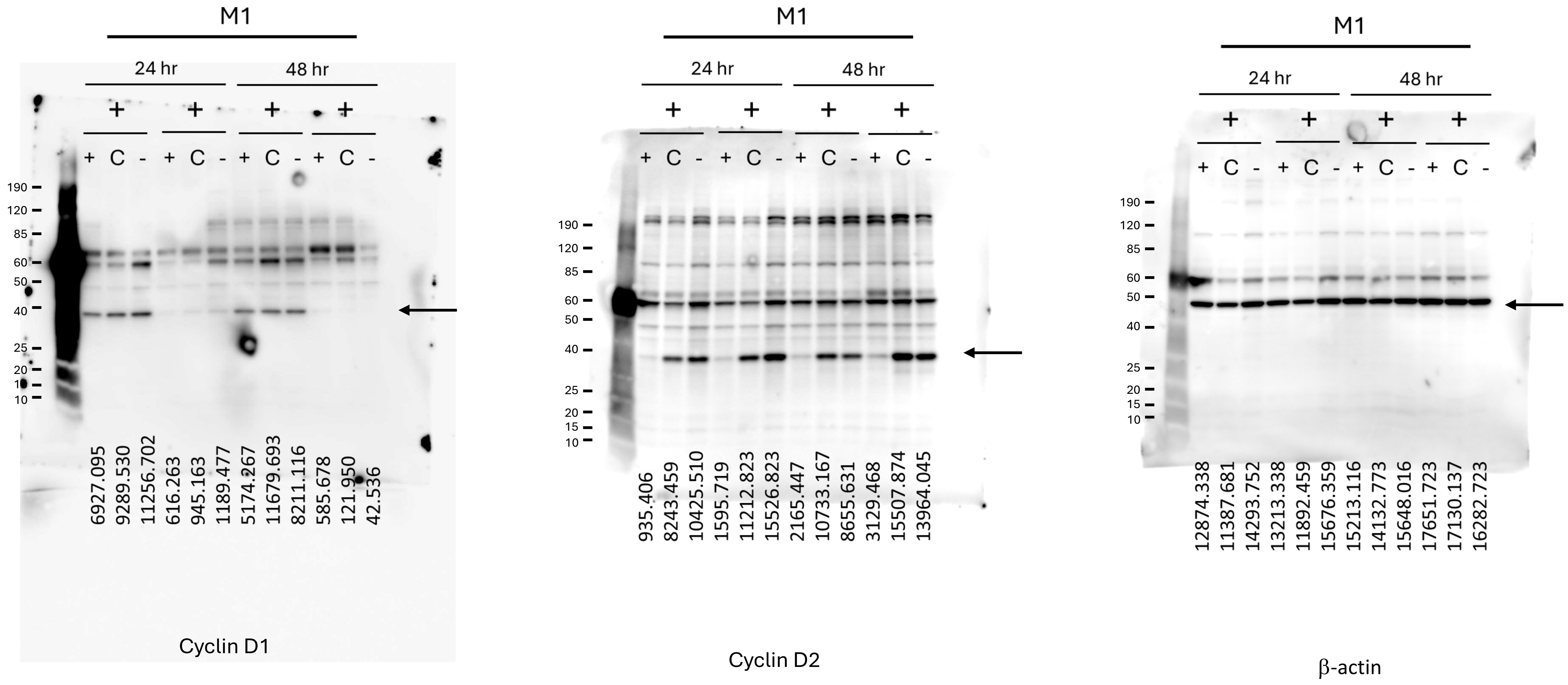
M1
24 hr
48 hr
+ C -
190
120
85
60
50
40
25
20
15
10
6927.095
9289.530
11256.702
616.263
945.163
1189.477
5174.267
11679.693
8211.116
585.678
121.950
42.536
Cyclin D1
935.406
8243.459
10425.510
1595.719
11212.823
15526.823
2165.447
10733.167
8655.631
3129.468
15507.874
13964.045
Cyclin D2
12874.338
11387.681
14293.752
13213.338
11892.459
15676.359
15213.116
14132.773
15648.016
17651.723
17130.137
16282.723
β-actin

Figure 4D

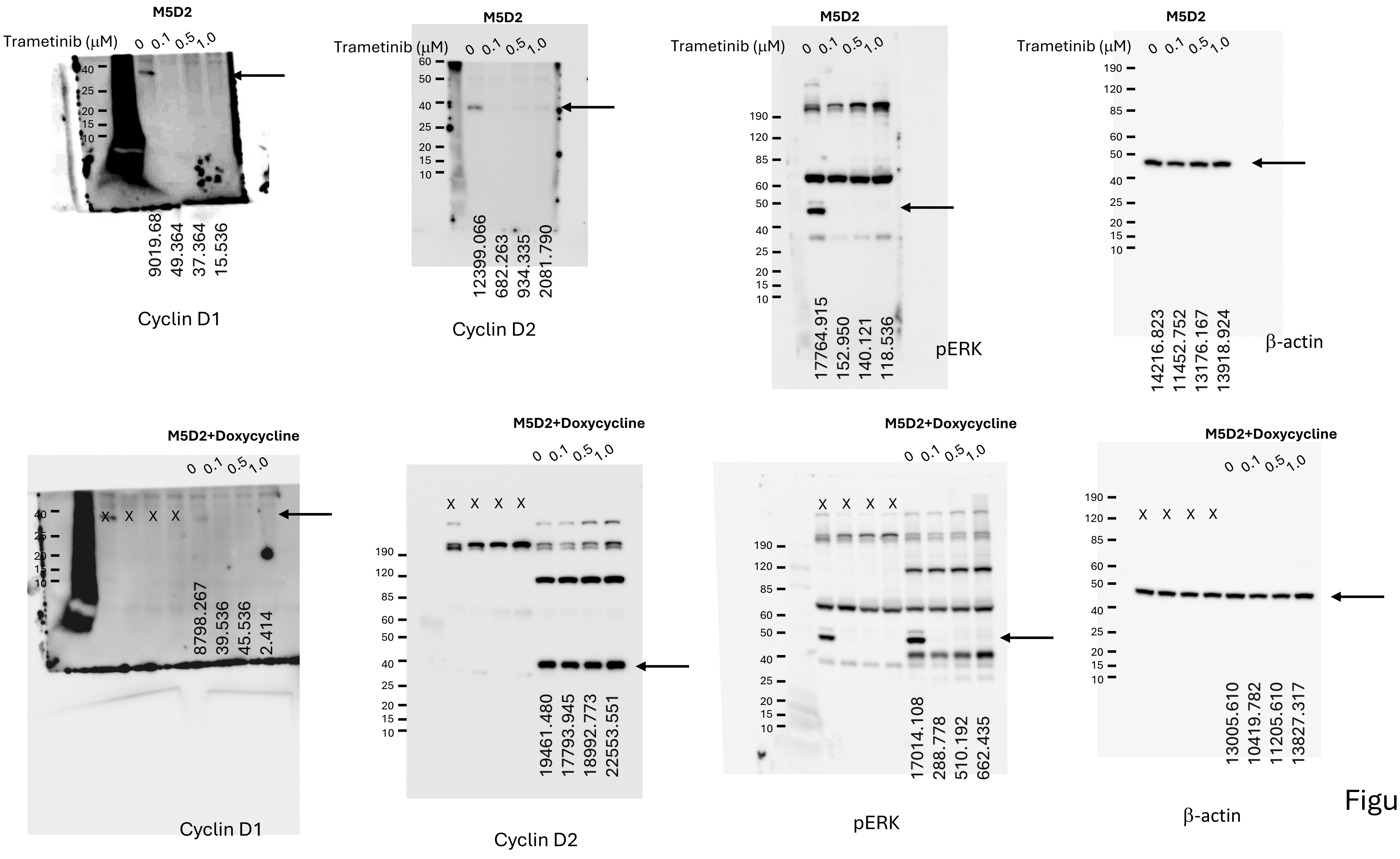
M5D2
Trametinib (μM) 0 0.1 0.5 1.0
40
25
20
15
10
9019.68
49.364
37.364
15.536
Cyclin D1
M5D2
Trametinib (μM) 0 0.1 0.5 1.0
60
50
40
25
20
15
10
12399.066
682.263
934.335
2081.790
Cyclin D2
M5D2
Trametinib (μM) 0 0.1 0.5 1.0
190
120
85
60
50
40
25
20
15
10
17764.915
152.950
140.121
118.536
pERK
M5D2
Trametinib (μM) 0 0.1 0.5 1.0
190
120
85
60
50
40
25
20
15
10
14216.823
11452.752
13176.167
13918.924
β-actin
M5D2+Doxycycline
0 0.1 0.5 1.0
X X X X
40
25
20
15
10
8798.267
39.536
45.536
2.414
Cyclin D1
M5D2+Doxycycline
0 0.1 0.5 1.0
X X X X
190
120
85
60
50
40
25
20
15
10
19461.480
17793.945
18992.773
22553.551
Cyclin D2
M5D2+Doxycycline
0 0.1 0.5 1.0
X X X X
190
120
85
60
50
40
25
20
15
10
17014.108
288.778
510.192
662.435
pERK
M5D2+Doxycycline
0 0.1 0.5 1.0
X X X X
190
120
85
60
50
40
25
20
15
10
13005.610
10419.782
11205.610
13827.317
β-actin

Figure 5A

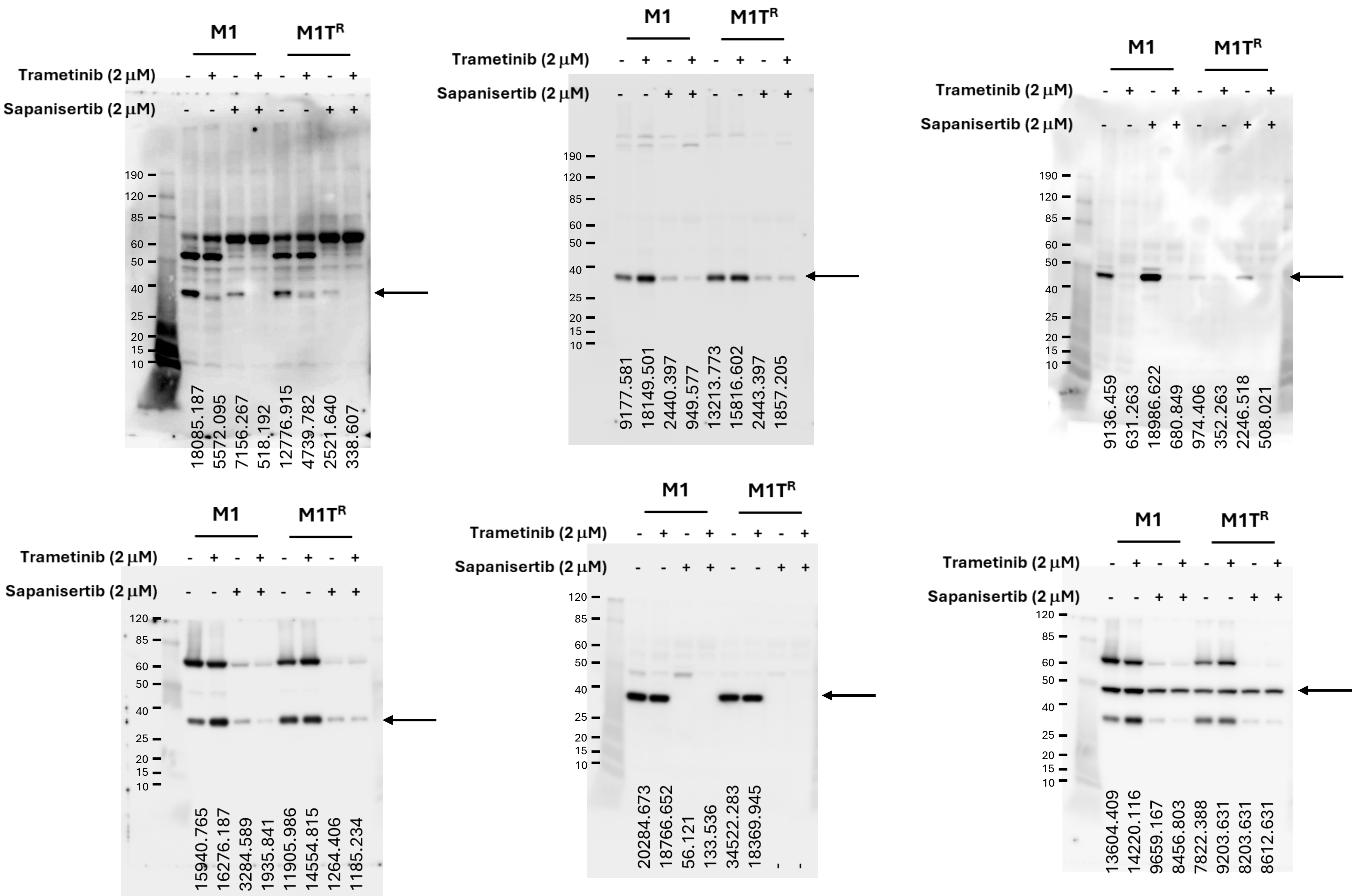
M1
M1T^R
Trametinib (2 μM) - + - + - + - +
Sapanisertib (2 μM) - - + + - - + +
190
120
85
60
50
40
25
20
15
10
18085.187
5572.095
7156.267
518.192
12776.915
4739.782
2521.640
338.607
M1
M1T^R
Trametinib (2 μM) - + - + - + - +
Sapanisertib (2 μM) - - + + - - + +
190
120
85
60
50
40
25
20
15
10
9177.581
18149.501
2440.397
949.577
13213.773
15816.602
2443.397
1857.205
M1
M1T^R
Trametinib (2 μM) - + - + - + - +
Sapanisertib (2 μM) - - + + - - + +
190
120
85
60
50
40
25
20
15
10
9136.459
631.263
18986.622
680.849
974.406
352.263
2246.518
508.021
M1
M1T^R
Trametinib (2 μM) - + - + - + - +
Sapanisertib (2 μM) - - + + - - + +
120
85
60
50
40
25
20
15
10
15940.765
16276.187
3284.589
1935.841
11905.986
14554.815
1264.406
1185.234
M1
M1T^R
Trametinib (2 μM) - + - + - + - +
Sapanisertib (2 μM) - - + + - - + +
120
85
60
50
40
25
20
15
10
20284.673
18766.652
56.121
133.536
34522.283
18369.945
-
-
M1
M1T^R
Trametinib (2 μM) - + - + - + - +
Sapanisertib (2 μM) - - + + - - + +
120
85
60
50
40
25
20
15
10
13604.409
14220.116
9659.167
8456.803
7822.388
9203.631
8203.631
8612.631

Figure 6B

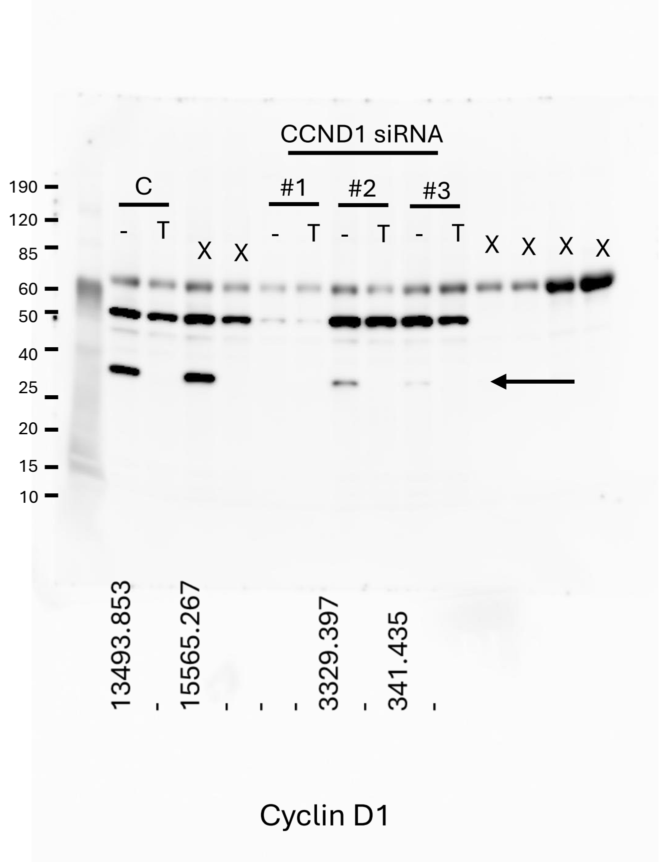
CCND1 siRNA
C
#1
#2
#3
- T X X - T - T - T X X X X
190
120
85
60
50
40
25
20
15
10
13493.853
15565.267
3329.397
341.435
Cyclin D1

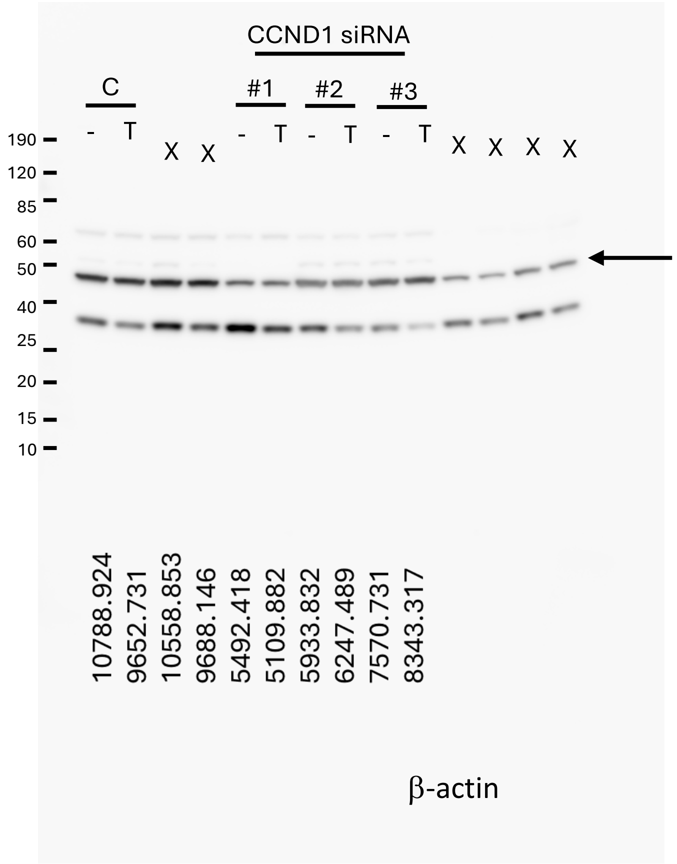
CCND1 siRNA
C
#1
#2
#3
- T X X - T - T - T X X X X
190
120
85
60
50
40
25
20
15
10
10788.924
9652.731
10558.853
9688.146
5492.418
5109.882
5933.832
6247.489
7570.731
8343.317
β-actin

Figure S1A

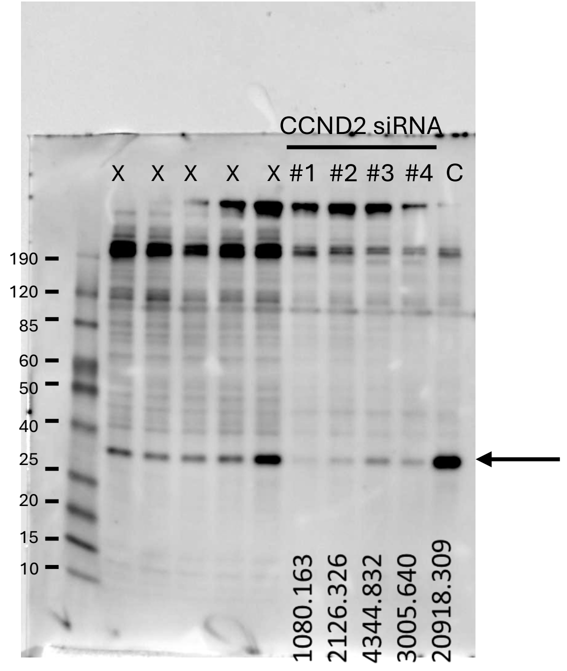

Cyclin D2

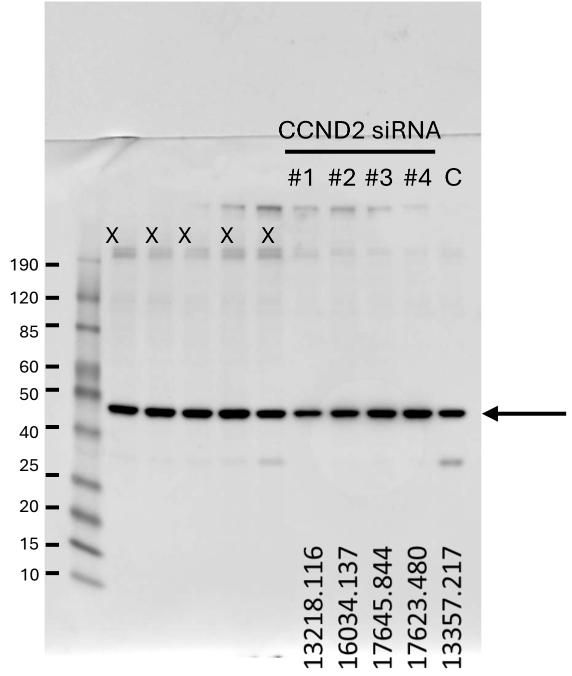

β-actin

Figure S1B

Supplemental Figure S3A (1)

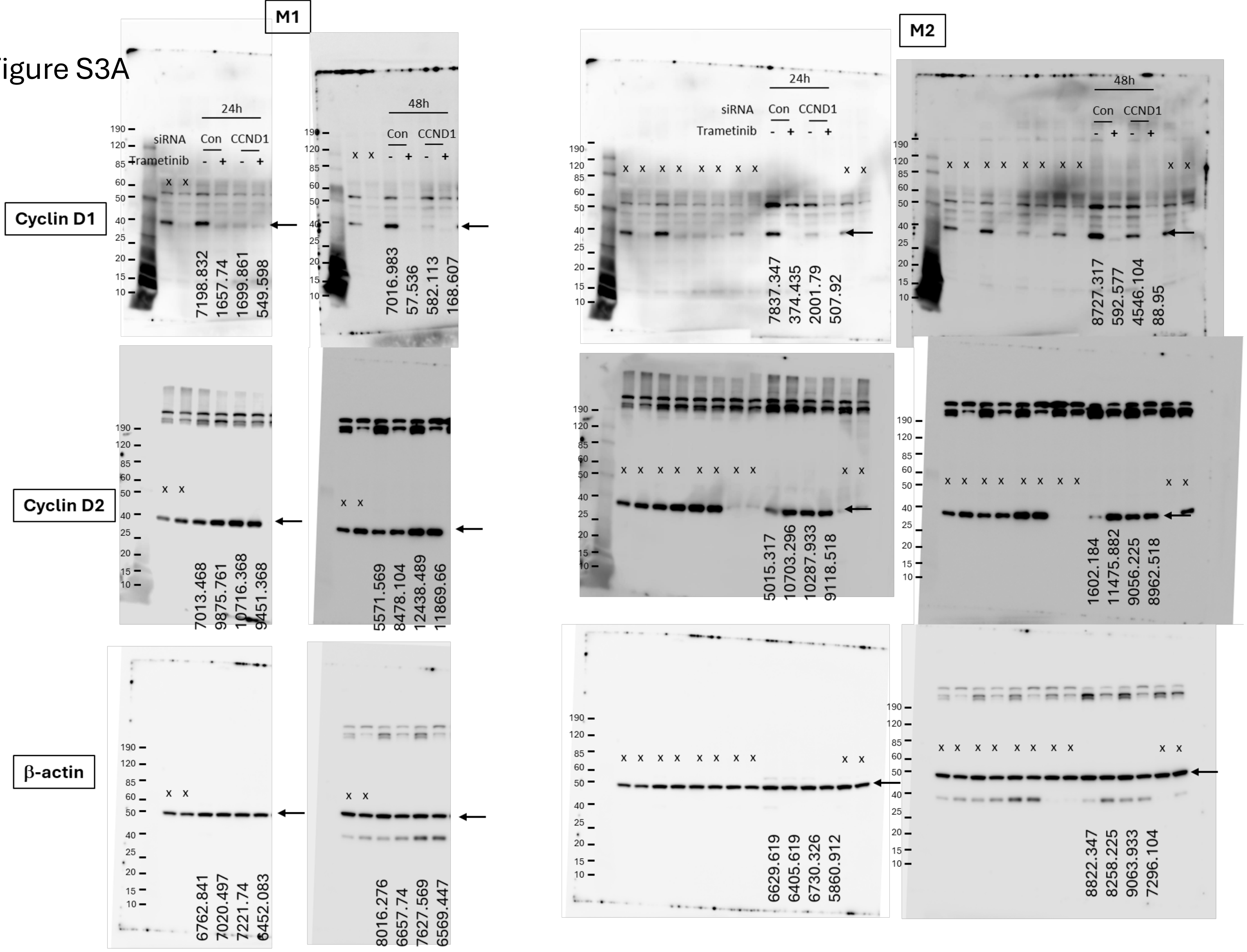

Supplemental Figure S3A (2)

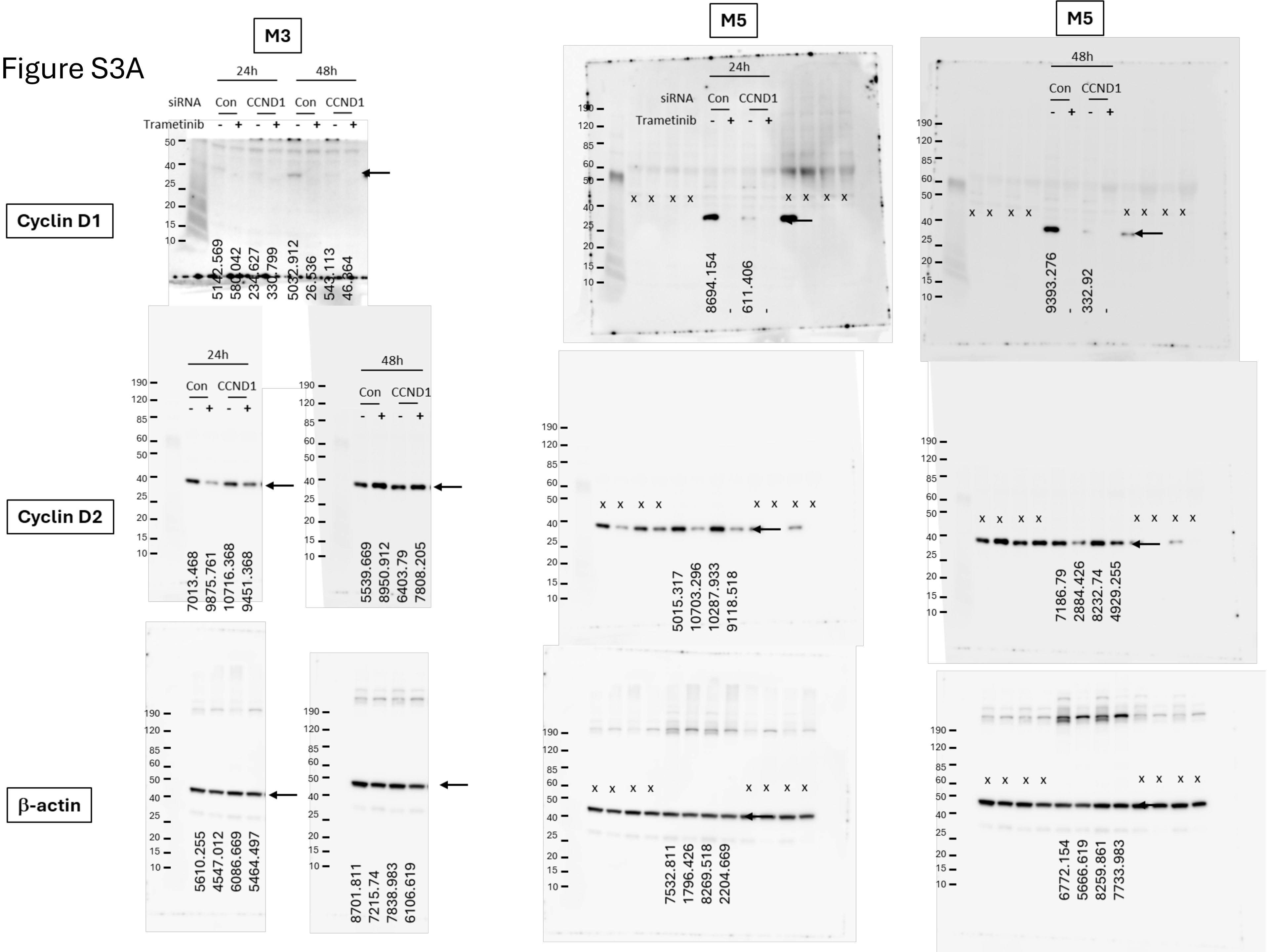

Supplemental Figure S3A (3)

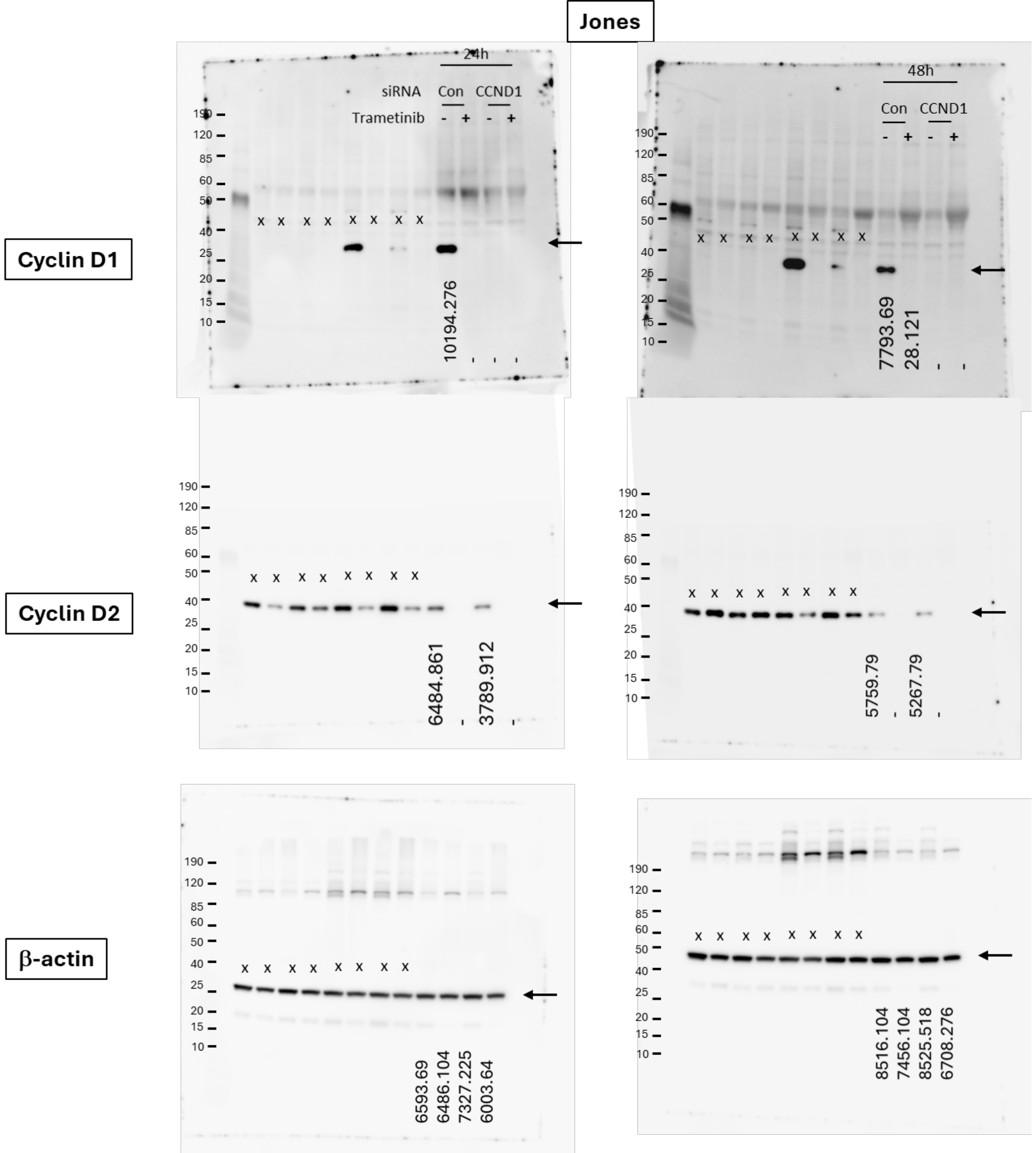

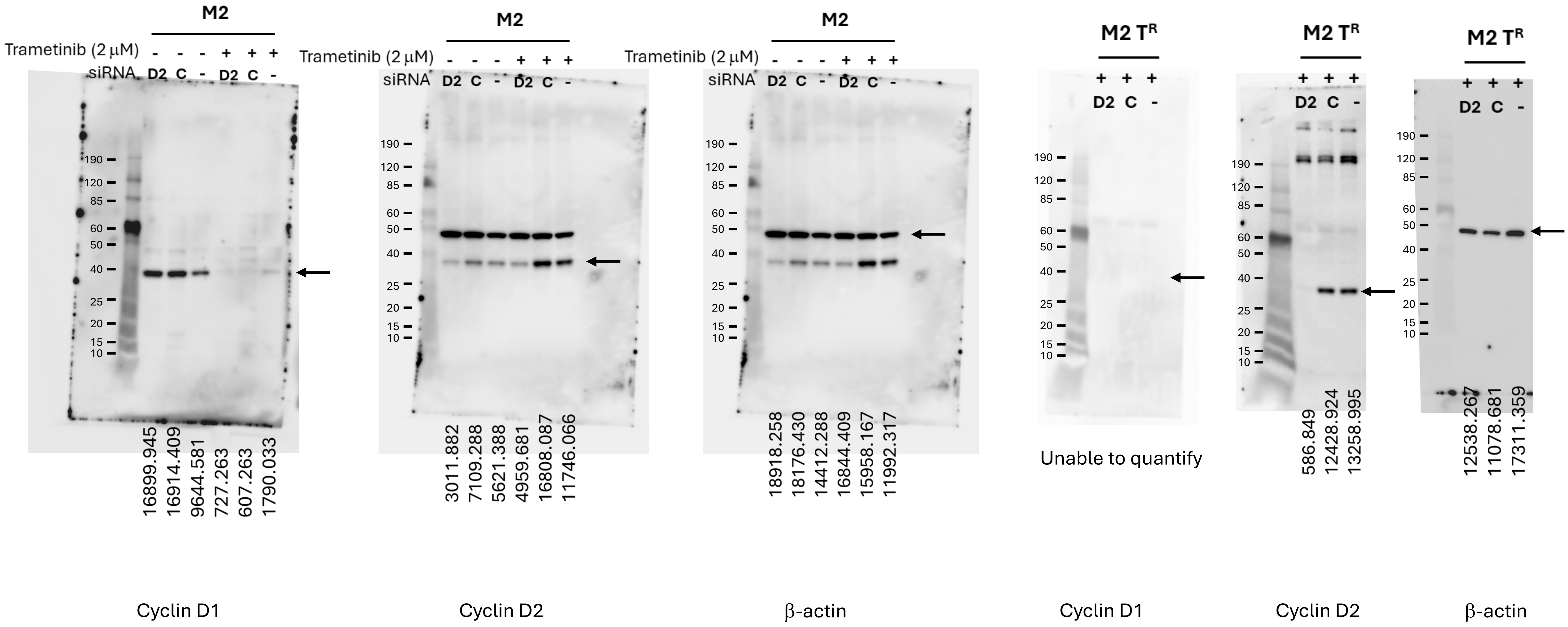
M2
Trametinib (2 μM) - - - + + +
siRNA D2 C - D2 C -
190
120
85
60
50
40
25
20
15
10
16899.945
16914.409
9644.581
727.263
607.263
1790.033
Cyclin D1
M2
Trametinib (2 μM) - - - + + +
siRNA D2 C - D2 C -
3011.882
7109.288
5621.388
4959.681
16808.087
11746.066
Cyclin D2
M2
Trametinib (2 μM) - - - + + +
siRNA D2 C - D2 C -
18918.258
18176.430
14412.288
16844.409
15958.167
11992.317
β-actin
M2 T^R
+ + +
D2 C -
Unable to quantify
Cyclin D1
M2 T^R
+ + +
D2 C -
586.849
12428.924
13258.995
Cyclin D2
M2 T^R
+ + +
D2 C -
12538.267
11078.681
17311.359
β-actin

Figure S4A

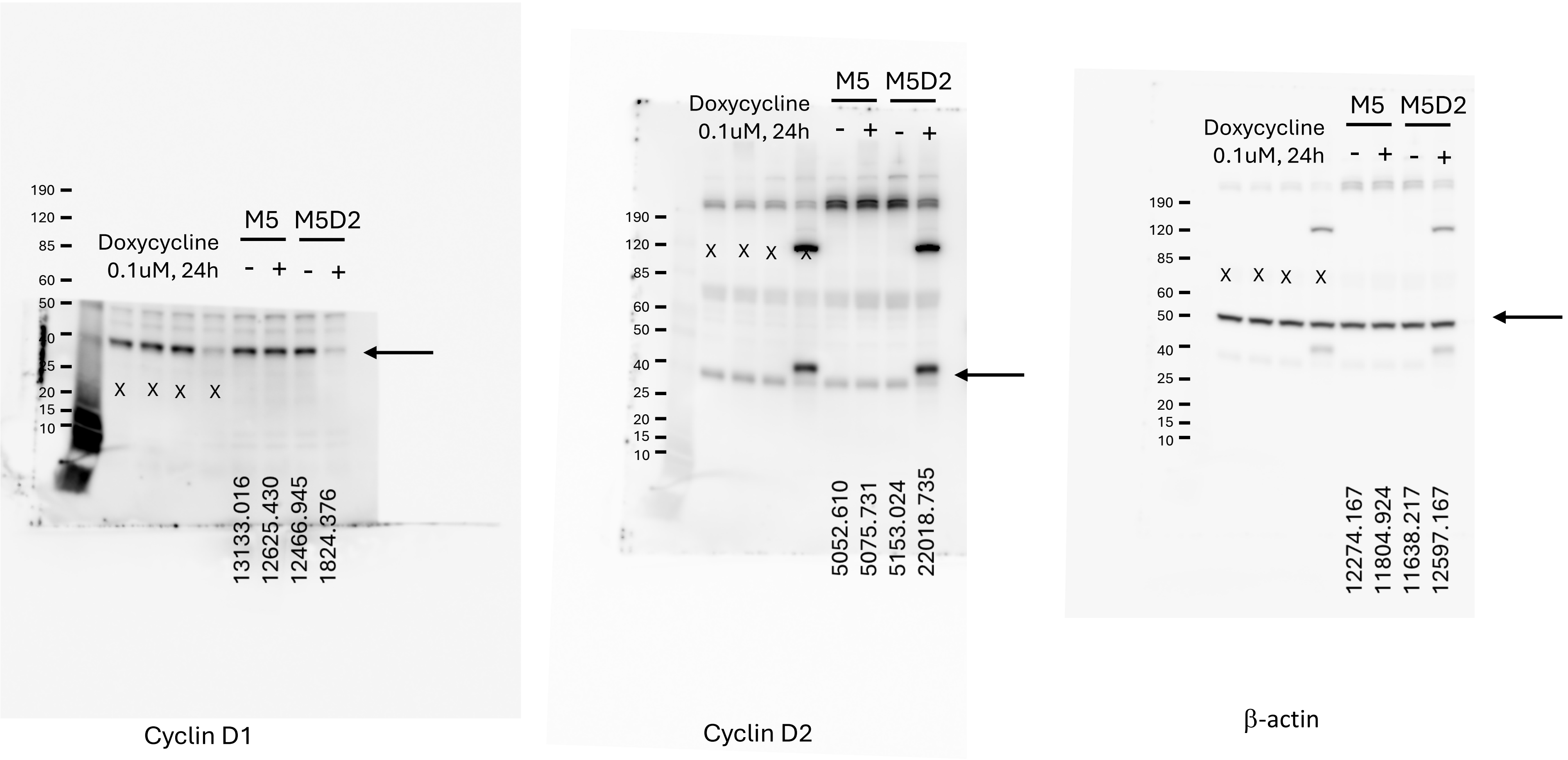
Doxycycline
0.1uM, 24h
M5
M5D2
- + - +
190
120
85
60
50
40
25
20
15
10
X X X X
13133.016
12625.430
12466.945
1824.376
Cyclin D1
Doxycycline
0.1uM, 24h
M5
M5D2
- + - +
190
120
85
60
50
40
25
20
15
10
X X X X
5052.610
5075.731
5153.024
22018.735
Cyclin D2
Doxycycline
0.1uM, 24h
M5
M5D2
- + - +
190
120
85
60
50
40
25
20
15
10
X X X X
12274.167
11804.924
11638.217
12597.167
β-actin

Figure S5B
