# Supplementary material for: Capacity for Compensatory Cyclin D2 Response Confers Trametinib Resistance in Canine Mucosal Melanoma
Source: Cancers (Basel). 2025 Jul 15;17(14):2357. doi: 10.3390/cancers17142357 (PMC12293520; doi:10.3390/cancers17142357)
Supplement: Supplementary file 1 [file cancers-17-02357-s001.zip › Wei BR et al 15-1 supplemental figures with legend_b.pptx]

## Slide 1
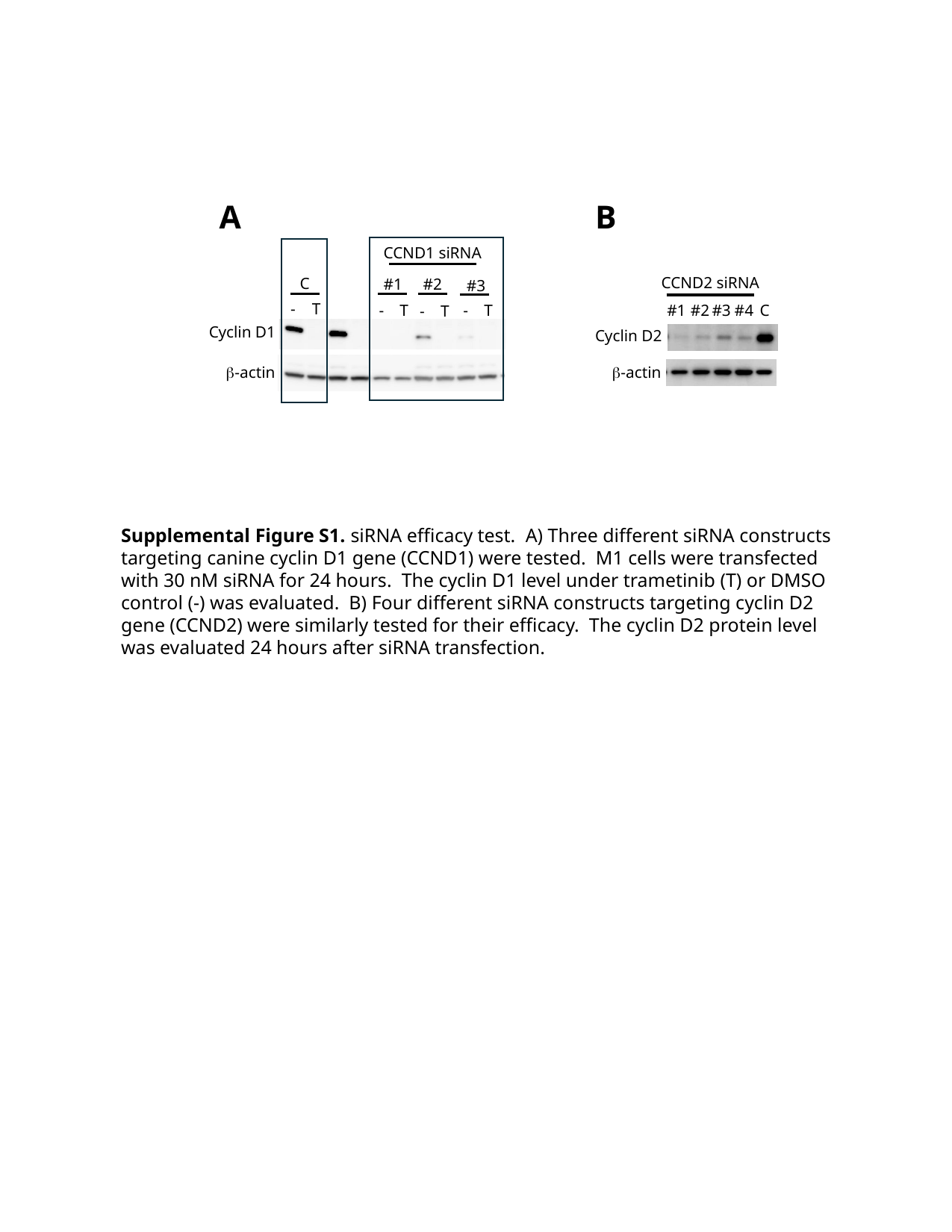

A
B
CCND1 siRNA
CCND2 siRNA
C
#1
#2
#3
-
T
#1
#2
#3
#4
C
-
T
-
T
-
T
Cyclin D1
Cyclin D2
b-actin
b-actin
Supplemental Figure S1. siRNA efficacy test. A) Three different siRNA constructs targeting canine cyclin D1 gene (CCND1) were tested. M1 cells were transfected with 30 nM siRNA for 24 hours. The cyclin D1 level under trametinib (T) or DMSO control (-) was evaluated. B) Four different siRNA constructs targeting cyclin D2 gene (CCND2) were similarly tested for their efficacy. The cyclin D2 protein level was evaluated 24 hours after siRNA transfection.

## Slide 2
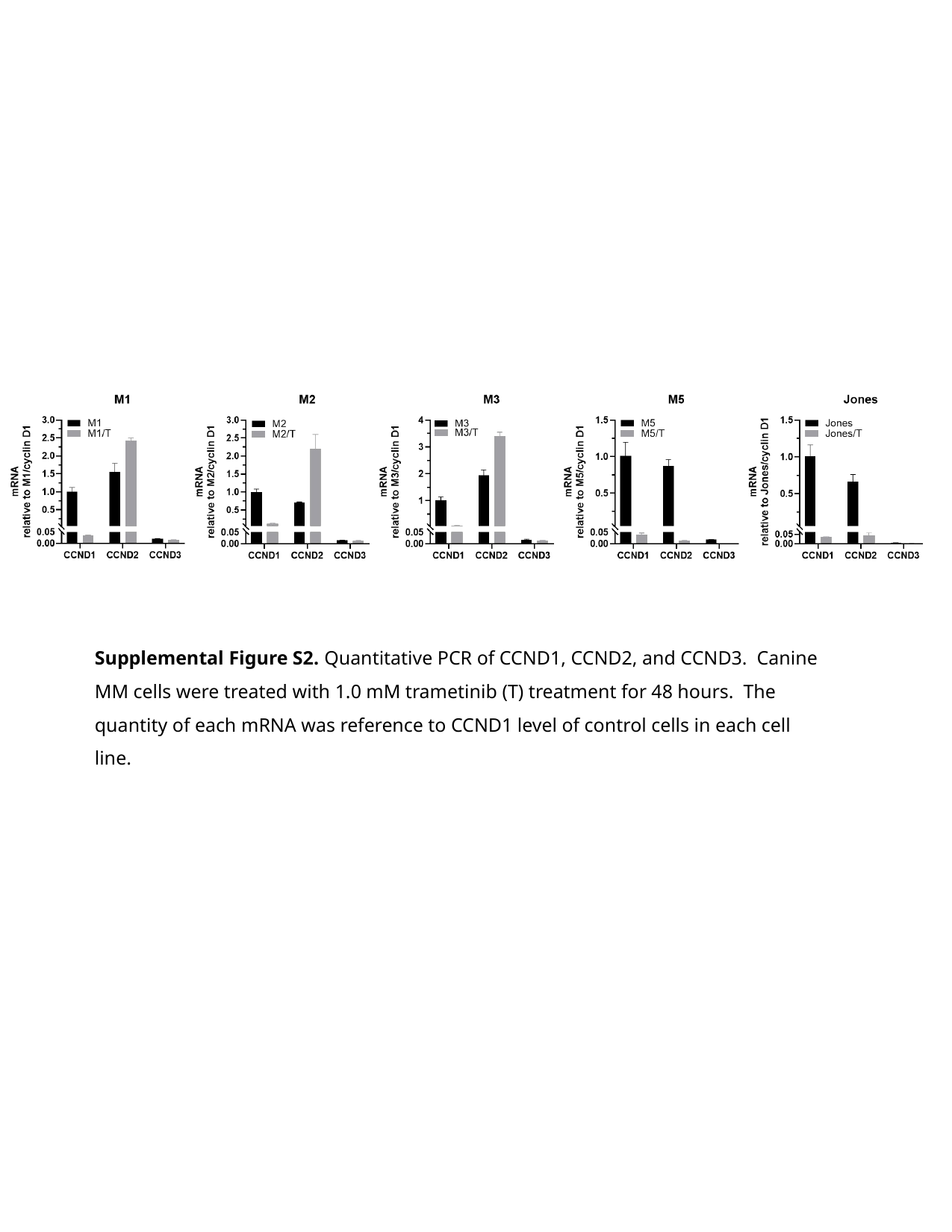

Supplemental Figure S2. Quantitative PCR of CCND1, CCND2, and CCND3. Canine MM cells were treated with 1.0 mM trametinib (T) treatment for 48 hours. The quantity of each mRNA was reference to CCND1 level of control cells in each cell line.

## Slide 3
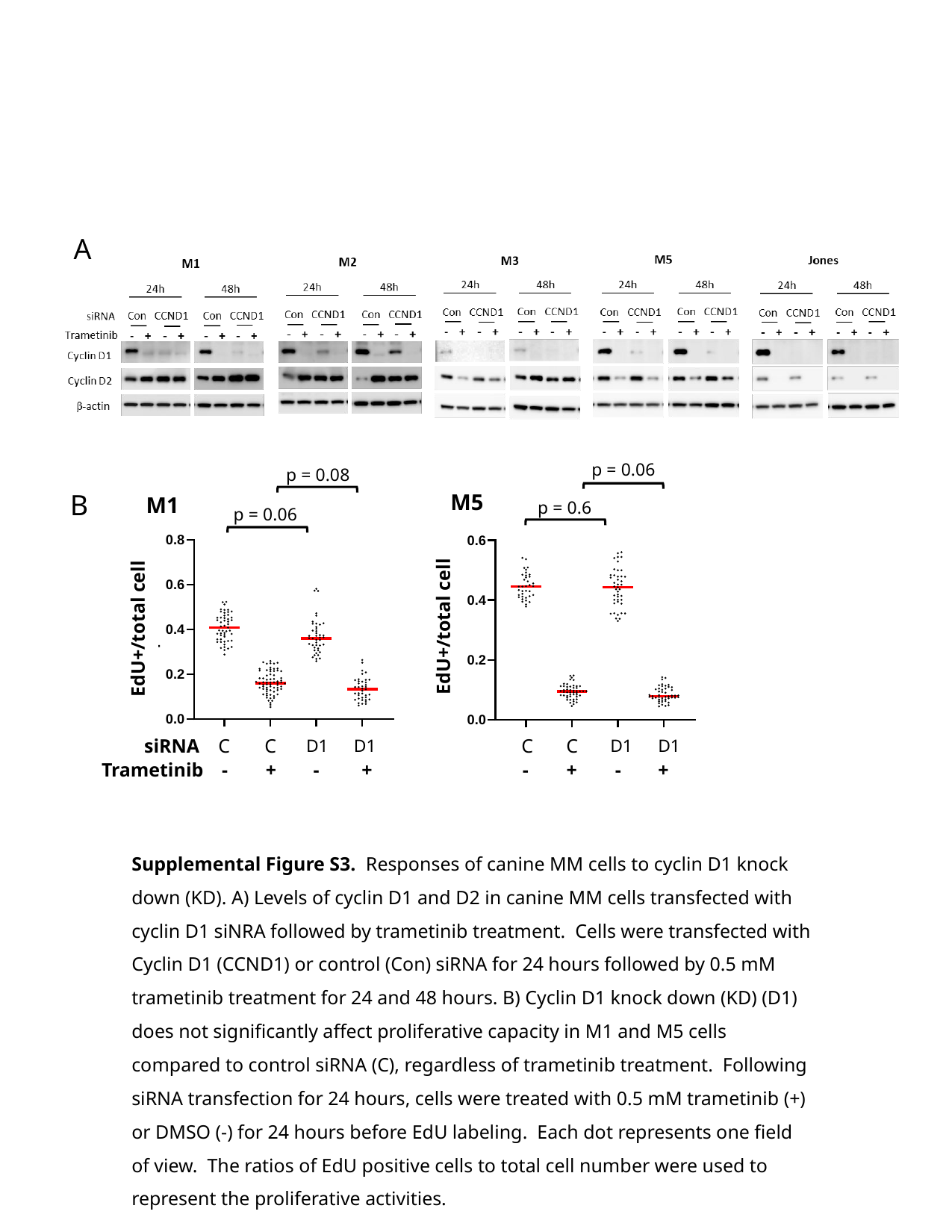

A
p = 0.06
p = 0.08
B
M5
M1
p = 0.6
p = 0.06
EdU+/total cell
EdU+/total cell
siRNA
C
C
C
C
D1
D1
D1
D1
Trametinib
-
+
-
+
-
+
-
+
Supplemental Figure S3. Responses of canine MM cells to cyclin D1 knock down (KD). A) Levels of cyclin D1 and D2 in canine MM cells transfected with cyclin D1 siNRA followed by trametinib treatment. Cells were transfected with Cyclin D1 (CCND1) or control (Con) siRNA for 24 hours followed by 0.5 mM trametinib treatment for 24 and 48 hours. B) Cyclin D1 knock down (KD) (D1) does not significantly affect proliferative capacity in M1 and M5 cells compared to control siRNA (C), regardless of trametinib treatment. Following siRNA transfection for 24 hours, cells were treated with 0.5 mM trametinib (+) or DMSO (-) for 24 hours before EdU labeling. Each dot represents one field of view. The ratios of EdU positive cells to total cell number were used to represent the proliferative activities.

## Slide 4
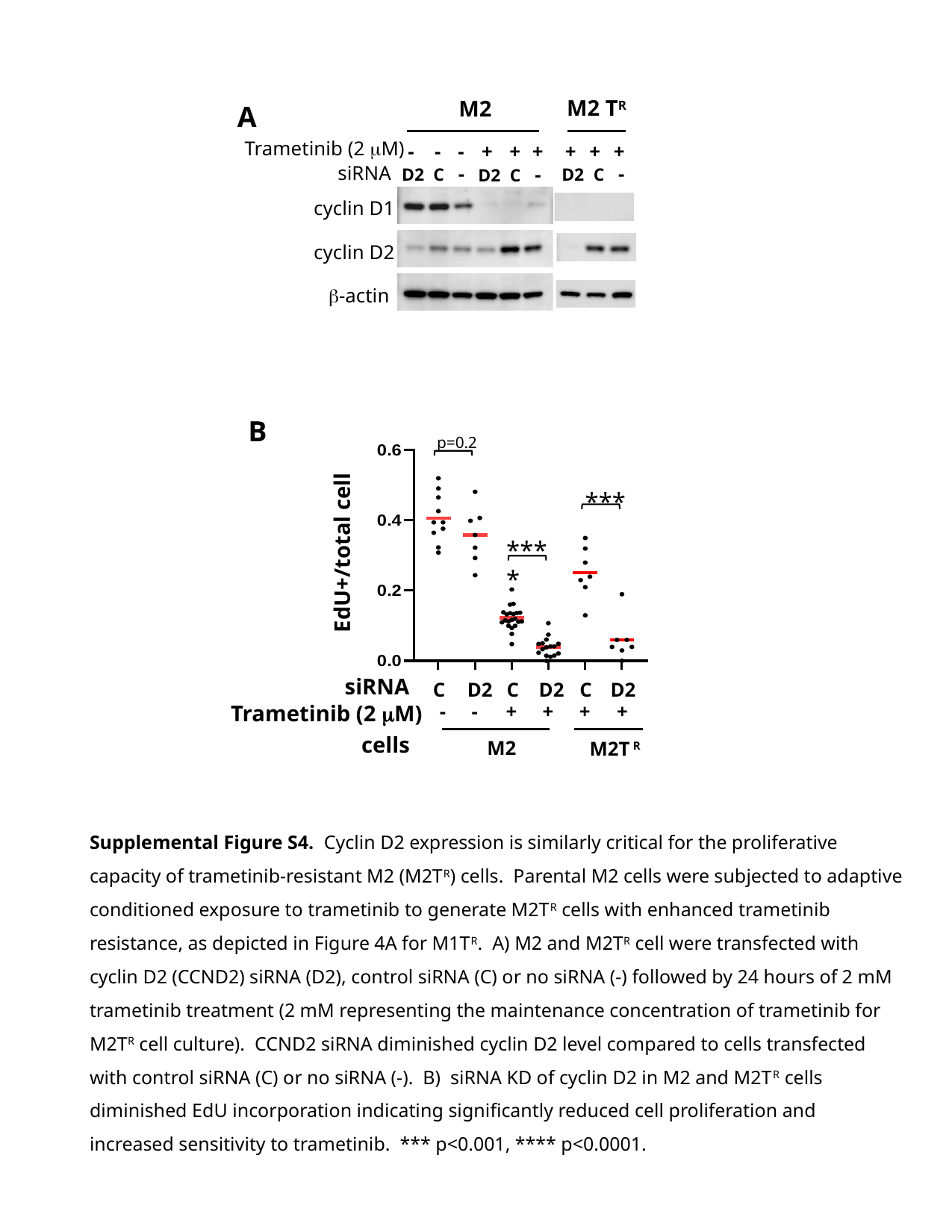

M2 TR
M2
A
Trametinib (2 mM)
-
-
-
+
+
+
+
+
+
siRNA
-
-
-
D2
C
D2
C
D2
C
cyclin D1
cyclin D2
b-actin
B
p=0.2
***
****
EdU+/total cell
siRNA
C
D2
C
D2
C
D2
Trametinib (2 mM)
-
-
+
+
+
+
cells
M2
M2T R
Supplemental Figure S4. Cyclin D2 expression is similarly critical for the proliferative capacity of trametinib-resistant M2 (M2TR) cells. Parental M2 cells were subjected to adaptive conditioned exposure to trametinib to generate M2TR cells with enhanced trametinib resistance, as depicted in Figure 4A for M1TR. A) M2 and M2TR cell were transfected with cyclin D2 (CCND2) siRNA (D2), control siRNA (C) or no siRNA (-) followed by 24 hours of 2 mM trametinib treatment (2 mM representing the maintenance concentration of trametinib for M2TR cell culture). CCND2 siRNA diminished cyclin D2 level compared to cells transfected with control siRNA (C) or no siRNA (-). B) siRNA KD of cyclin D2 in M2 and M2TR cells diminished EdU incorporation indicating significantly reduced cell proliferation and increased sensitivity to trametinib. *** p<0.001, **** p<0.0001.

## Slide 5
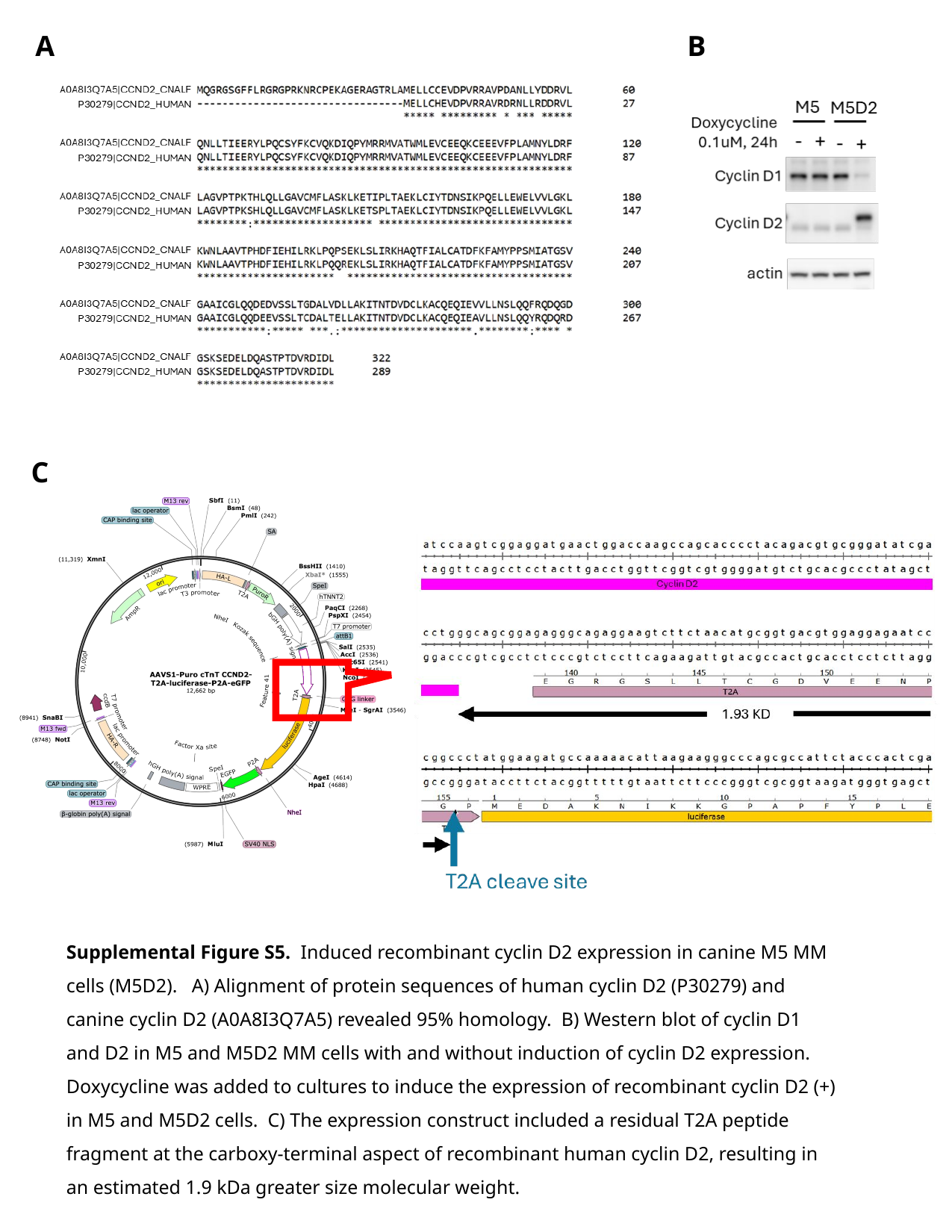

A
B
C
Supplemental Figure S5. Induced recombinant cyclin D2 expression in canine M5 MM cells (M5D2). A) Alignment of protein sequences of human cyclin D2 (P30279) and canine cyclin D2 (A0A8I3Q7A5) revealed 95% homology. B) Western blot of cyclin D1 and D2 in M5 and M5D2 MM cells with and without induction of cyclin D2 expression. Doxycycline was added to cultures to induce the expression of recombinant cyclin D2 (+) in M5 and M5D2 cells. C) The expression construct included a residual T2A peptide fragment at the carboxy-terminal aspect of recombinant human cyclin D2, resulting in an estimated 1.9 kDa greater size molecular weight.

## Slide 6
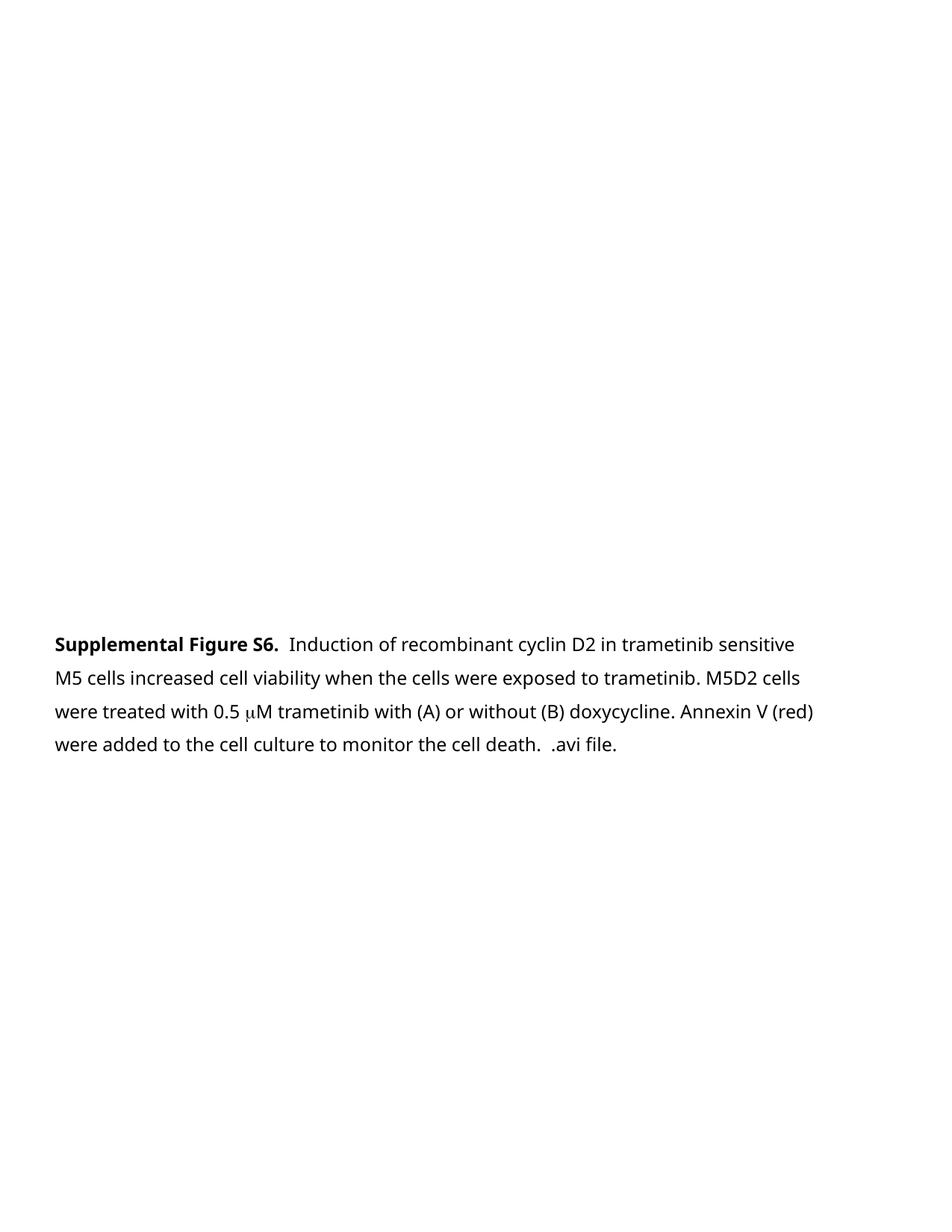

Supplemental Figure S6. Induction of recombinant cyclin D2 in trametinib sensitive M5 cells increased cell viability when the cells were exposed to trametinib. M5D2 cells were treated with 0.5 mM trametinib with (A) or without (B) doxycycline. Annexin V (red) were added to the cell culture to monitor the cell death. .avi file.
